# Differential Redox State Contributes to Sex Disparities in the Response to Influenza Virus Infection in Male and Female Mice

**DOI:** 10.3389/fimmu.2018.01747

**Published:** 2018-07-30

**Authors:** Ignacio Celestino, Paola Checconi, Donatella Amatore, Marta De Angelis, Paolo Coluccio, Rosanna Dattilo, Danilo Alunni Fegatelli, Ann Maria Clemente, Paola Matarrese, Maria Gabriella Torcia, Romina Mancinelli, Caterina Loredana Mammola, Enrico Garaci, Anna Rita Vestri, Walter Malorni, Anna Teresa Palamara, Lucia Nencioni

**Affiliations:** ^1^Department of Public Health and Infectious Diseases, Pasteur Institute Cenci Bolognetti Foundation, Sapienza University of Rome, Rome, Italy; ^2^San Raffaele Pisana, IRCCS, Telematic University, Rome, Italy; ^3^Department of Hematology, Oncology and Molecular Medicine, Istituto Superiore di Sanità, Rome, Italy; ^4^Department of Clinical and Experimental Medicine, University of Florence, Florence, Italy; ^5^Center for Gender-Specific Medicine, Istituto Superiore di Sanità, Rome, Italy; ^6^Department of Anatomical, Histological, Forensic Medicine and Orthopedic Sciences, Sapienza University of Rome, Rome, Italy

**Keywords:** sex differences, gender, hormones, influenza virus, redox state, glutathione

## Abstract

Influenza virus replicates intracellularly exploiting several pathways involved in the regulation of host responses. The outcome and the severity of the infection are thus strongly conditioned by multiple host factors, including age, sex, metabolic, and redox conditions of the target cells. Hormones are also important determinants of host immune responses to influenza and are recently proposed in the prophylaxis and treatment. This study shows that female mice are less susceptible than males to mouse-adapted influenza virus (A/PR8/H1N1). Compared with males, PR8-infected females display higher survival rate (+36%), milder clinical disease, and less weight loss. They also have milder histopathological signs, especially free alveolar area is higher than that in males, even if pro-inflammatory cytokine production shows slight differences between sexes; hormone levels, moreover, do not vary significantly with infection in our model. Importantly, viral loads (both in terms of viral M1 RNA copies and tissue culture infectious dose 50%) are lower in PR8-infected females. An analysis of the mechanisms contributing to sex disparities observed during infection reveals that the female animals have higher total antioxidant power in serum and their lungs are characterized by increase in (i) the content and biosynthesis of glutathione, (ii) the expression and activity of antioxidant enzymes (peroxiredoxin 1, catalase, and glutathione peroxidase), and (iii) the expression of the anti-apoptotic protein Bcl-2. By contrast, infected males are characterized by high expression of NADPH oxidase 4 oxidase and phosphorylation of p38 MAPK, both enzymes promoting viral replication. All these factors are critical for cell homeostasis and susceptibility to infection. Reappraisal of the importance of the host cell redox state and sex-related effects may be useful in the attempt to develop more tailored therapeutic interventions in the fight against influenza.

## Introduction

Viruses replicate in the living cells of their hosts and use many intracellular pathways for their own advantage. Consequently, host factors like age, general health, metabolic, and redox conditions of the cells can have important repercussions on different steps of the virus life cycle (1–4). Furthermore, general redox state may also affect host immune response to viral replication (5–8). Cells containing high levels of thiols, e.g., glutathione (GSH) and cysteine content, or characterized by higher antioxidant defenses, as well as abundant expression of Bcl-2 proteins family, are less permissive to viral replication, including influenza (2). Moreover, our and other groups previously demonstrated that cells infected with influenza virus were characterized by low levels of GSH (2, 9–12) and by an increase of reactive oxygen species (ROS) production (10, 11, 13). During influenza virus infection, there is also a depletion of key antioxidant enzymes, due to their secretion or because of virus-induced loss of lung cells (14, 15). The oxidative stress is useful for the virus since many pathways involved in the regulation of viral replication and host responses to viral infection are highly responsive to even transient changes in the redox state of the cytoplasmic environment (16). In fact, some enzymes like protein disulfide isomerase (PDI) or NADPH oxidase 4 (NOX4) regulate specific steps of virus life cycle, including the folding and maturation of viral glycoprotein hemagglutinin (10) and the nuclear-cytoplasmic export of viral nucleoprotein (NP) (11, 17, 18).

Sex and gender, that refer to biology and behavior, respectively (19), also impact viral infections. Analysis of several epidemiological studies has highlighted that disease severity and fatality following exposure to influenza A viruses are generally higher in women than men (19, 20). The mechanisms underlying this sex/gender difference are several and tightly interconnected; behavioral, immunological, hormonal, and genetic factors are all included (20). Focusing on biological factors, it is known that females mount a higher immune response than males, which can accelerate virus clearance and reduce virus load, but can also make females more prone to immunopathology and to development of autoimmune disease (21). Klein et al. (20, 22, 23) reported that the exaggerated immunity and consequent immunopathology lead females to greater morbidity and mortality with respect to males. Such a response can be modulated by hormone concentrations, and so age may also affect the sex-related variability (24, 25). Epidemiological studies in which results were stratified by age in fact, report that hospitalization and morbidity rates due to influenza A viruses are higher in males than in females from birth to 15–19 years (26–31). Non-endocrine factors, as genetic ones, could prevail in the latter case. It has been shown that genetic variation in chromosome Y regulates susceptibility to influenza A virus, making specific variants in males mice more susceptible to infection (32).

Interesting parameters that also differently characterize cells isolated from male and female animals were the redox ones (33, 34). Malorni et al. (35) reported differences between vascular smooth muscle cells (VSMC) from male and female rats in terms of “basal” redox balance. Either H_2_O_2_ or ${\text{O}}_{2}^{-}$ levels were significantly lower in VSMC from females than those from male rats. Moreover, the intracellular GSH content was higher in female than in male rats. The same authors found that antioxidant enzyme activity was significantly higher in VSMC from female than in male, independently from the stimuli that induced stress (35, 36). Many redox-sensitive cell-signaling pathways are differently activated in both sexes (37).

On the basis of this evidence, in this study, we verified the hypothesis that host redox state plays a role in sex disparities in the outcome of influenza virus infection. To evaluate viral replication in male and female mice, we chose the Balb/c strain, which is considered a Th2-type strain (38), to better highlight the effect of the virus (as opposed to the immune response). Female and male mice were infected with a mouse-adapted strain of influenza A (H1N1) and the progression of disease was monitored by measuring some redox parameters usually altered during infection. We found that in terms of both survival and clinicopathological parameters of disease, the female mice displayed higher resistance to the infection, due to significant differences in the systemic and pulmonary “redox profiles” between female and male mice.

## Materials and Methods

In accordance with national law, the experiments described in this manuscript were approved by the Italian Ministry of Health, which verified the ethical and scientific appropriateness of the research. All animals received humane treatment, and every effort was made to minimize their suffering. Unless otherwise stated, all commercial products cited were used in accordance with the manufacturers’ instructions.

### Mice and Virus Infection

Balb/c 6-week-old mice [400 females, body weight (bw) range = 15–19 g; 400 males, bw = 19–23 g] were purchased from Harlan Laboratories (Milan, Italy). Animals were housed under specific pathogen-free conditions (5/cage, SmartFlow IVC Rack, Tecniplast, Varese, Italy) at 12:12 h light:dark cycle, and *ad libitum* access to food and water. After 1 week, each mouse was individually weighed and randomly assigned to an experimental group.

A mouse-adapted strain of influenza A/Puerto Rico/8/34 (H1N1; PR8) was used. In our experiments, 1 plaque-forming unit (PFU) of PR8 stock was equivalent to 2.9 × 10^3^ genome copies, approximately 2.0 × 10^3^ genome copies/tissue culture infectious dose 50% (TCID_50_) according to the relationship between TCID_50_ and PFU provided by the American Type Culture Collection. The 50% mouse lethal dose (MLD_50_) was determined in female and male mice that had been lightly anesthetized by isofluorane (Esteve, Milan, Italy) inhalation and intranasally inoculated with PR8 at different doses (0.01–10 PFU/animal).

For assessment of morbidity and survival related to seasonal-like influenza infections, the *inoculum* consisted of 50 µl of sterile phosphate-buffered saline (PBS), alone (mock-infected controls) or containing 0.5 PFU/mouse of PR8 (infected animals).

Infected and control animals were daily monitored up to 21 days post-infection (p.i.). Each animal was weighed, its rectal temperature was measured (Temp Thermocouple Meter, Oakton, USA), and the clinical severity of disease was scored using the following scale (39, 40): 0 = no visible signs of disease; 1 = slight ruffling of fur; 2 = ruffled fur, reduced mobility; 3 = ruffled fur, reduced mobility, rapid breathing; 4 = ruffled fur, minimal mobility, huddled appearance, rapid and/or labored breathing indicative of pneumonia.

At the end of the experiments, the mice were euthanized with an overdose of tiletamine/zolazepam (Virbac, Milan, Italy) (800 mg/kg bw) and xylazine (Bayer, Milan, Italy) (100 mg/kg bw). Specimens for analysis [blood, broncho-alveolar lavage fluid (BALF), and lungs] were then collected as described below.

### Blood

#### Serum Total Antioxidant Capacity (TAC) Assay

On p.i. days 3, 6, 9, and 21, blood was collected from the retro-orbital venous sinuses of control and PR8-infected mice. The recovery was made with a Pasteur pipette after ocular instillation of oxybuprocaine (1 drop/eye) (Novartis, Siena, Italy). The sample was allowed to clot for 45 min (to facilitate removal of all platelets and precipitates) and then centrifuged at 10,000 × *g* for 15 min at +4°C. The serum was stored at −80°C prior to assay with the TAC Kit (JaICA, Florence, Italy), which measures the sample’s capacity to convert Cu^+2^ to Cu^+1^.

#### Sex Hormone Quantification

Testosterone and estradiol quantification was performed using a colorimetric competitive enzyme immunoassay kit purchased from Enzo Life Sciences (3V Chimica, Rome, Italy), according to the manufacturers’ instructions.

### Broncho-Alveolar Lavage Fluid (BALF)

Mice were euthanized, and a sterile 23-G catheter was inserted into the exposed tracheal lumen. Two instillations of sterile PBS (0.8 ml) containing protease inhibitors (Sigma-Aldrich, Milan, Italy) were injected through the catheter and aspirated as previously described (41). The BALF samples were centrifuged at 1,000 × *g* for 15 min at +4°C and the supernatant stored at −80°C prior to analysis.

#### Total Protein Content

For assessment of lung damage, the total protein content of each BALF specimen was measured with a standard Micro BCA Kit (Pierce, Monza, Italy). BALF samples (150 µl) were pipetted into a microplate well, working reagent (150 µl) was added, and the plate was incubated at 37°C for 2 h and cooled to room temperature. The optical density of each solution was measured at 570 nm with a Multiskan Ex Reader (Thermo Fisher Scientific, Monza, Italy).

#### Cytokine Quantification

A multiplex assay was used to measure cytokine (IL-1, IL-6, TNF-α, IL-10, IFN-γ, CCL2-MCP1, and CCL3-MIP1) levels in each BALF sample. Plates were read on a Bio-Plex MAGPIX instrument, and data were analyzed with Bio-Prosoftware (Bio-Rad, Milan, Italy).

### Lungs

#### Assay of Viral Titers

Whole lungs isolated from infected female and male mice were removed, weighed, frozen, and stored at −80°C. For the quantification of viral M1 RNA copies, total RNA was extracted from thawed lungs that had been homogenized in TRI Reagent (Sigma-Aldrich, Milan, Italy) (1 ml/75 mg of tissue) with a Polytron homogenizer. The RNA pellet was washed with 1 ml of 75% ethanol (7,500 × *g* for 5 min at +4°C) and air-dried for 30 min. Diethylpyrocarbonate water (100 µl) was added, and tube was heated to 55°C for 15 min to facilitate dissolution. The isolated RNA was treated with DNase I (Invitrogen, Life Technologies, Monza, Italy), and its quality and quantity were verified spectrophometrically (Pearl Nanophotometer, IMPLEN, Munich, Germany). The number of viral M1 RNA copies was determined by quantitative real time RT-PCR using the One Step Influenza A/B r-gene and Quanti FluA kits (BioMérieux, Florence, Italy). For the evaluation of TCID_50_, lungs were homogenized in RPMI 1640 medium, and homogenates were subjected to TCID_50_ assay on MDCK cells. The number of wells showing positive cytopathic effects was scored, and the titer was calculated as previously described (42).

#### Histologic Examination

Lung histology was evaluated in female and male infected mice (*n* = 25/group). Mock-infected mice were used as controls. Mice were sacrificed at 3, 6, 9, and 21 days p.i. Each sacrifice was followed by complete necroscopy with macroscopic and microscopic examinations of the lungs.

For the histopathological and morphological examination, each lung was fixed in buffered formalin at room temperature for 48 h and embedded in paraffin with a melting point of 55–57°C. Sections (3-μm thick) were stained with hematoxylin and eosin and Masson’s trichrome.

The samples were evaluated independently and blindly by three investigators (Caterina Loredana Mammola, Antonio Franchitto, and Romina Mancinelli), and necroinflammatory changes were scored as follows (43, 44): 0 = no lesions; 1 = mild focal inflammation; 2 = moderate–severe inflammation or necrosis affecting less than 25% of lung tissue examined; 3 = severe inflammation with necrosis or severe inflammation affecting 25–50% of lung tissue examined; 4 = severe inflammation with necrosis affecting more than 50% of the lung tissue examined. For each lung, at least five slides were analyzed. Briefly, serial paraffin sections were obtained per animal. For each sample, 10 fields were analyzed per section. Alveoli were identified and bordered to calculate the corresponding areas. All ambiguous structures, airways, and vascular structures were excluded. The tissue and airspace areas were tabulated using the IAS Delta Sistemi software (Rome, Italy) (10, 45–47).

#### Assays of Thiols Levels and Antioxidant Enzyme Activities

A sterile 23-G butterfly needle was inserted into the euthanized mouse’s right ventricle and connected to a peristaltic pump (Generalcontrol, Milan, Italy). The lungs were then perfused with PBS containing 50 U/ml heparin (Sigma-Aldrich, Milan, Italy) to remove erythrocytes and clots. Cuts were made in the liver to facilitate perfusate outflow. The lungs were then removed, weighed, frozen in liquid nitrogen, and stored at −80°C until assayed.

Intracellular glutathione (GSH) and oxidized forms [oxidized glutathione (GSSG)] were measured in lung homogenates with the Glutathione Assay Kit (Cayman Chemical, Florence, Italy) following the manufacturer’s instructions, after deproteinization with metaphosphoric acid of the samples. For GSSG quantification, an aliquot of deproteinized samples was first incubated with 2-vinylpyridine to derivatize GSH. Reduced GSH levels were obtained by differences between total GSH and GSSG.

The total amount of free thiols in deproteinized samples from lung homogenates and in serum were measured by a standard colorimetric assay using Ellman’s reagent (48).

Catalase (CAT), superoxide dismutase (SOD), and glutathione peroxidase (GSHPx) activities were also measured with specific kits (Cayman Chemical, Florence, Italy). Calculation of enzymatic activity was determined following the manufacturer’s instructions.

#### RT-PCR Analysis of Pulmonary mRNA Levels

Total RNA was isolated from the lungs as described above and used as a template for generating cDNA (iScript cDNA Synthesis Kit, Bio-Rad, Milan, Italy). An aliquot of the cDNA was subjected to 40 cycles of RT-PCR amplification (95°C, 10 s; 60°C, 30 s) using iQ SYBR Green Supermix and a LightCycler iQ 5 (Bio-Rad, Milan, Italy). To ensure that the primers produced a single and specific PCR amplification product, a melting curve analysis was carried out at the end of the PCR cycle. The housekeeping genes glucuronidase beta (Gusb), ribosomal protein L13A (Rpl13a), and glyceraldehyde-3-phosphate dehydrogenase (Gapdh) were used for normalization. Relative quantitative evaluation was performed by the comparative ΔΔCt method.

The following forward and reverse primers were used: glutathione reductase (GR) (TTCAGTTGGCATGTCATC forward; CCGTGGATAATTTCTATGTGA reverse), glutathione synthase (GSS) (GTGCTACTGATTGCTCAA forward; ACATGGATCTTCCTGTCT reverse), glutamate cysteine ligase (GCL) (AAGTCCCTCTTCTTTCCA forward; CCTTGAATATTGGCACATTG reverse), Bcl-2 (CCTACGGATTGACATTCTC forward; ATACATAAGGCAACCACAC reverse), Rpl13a (ATGGGATGAATCAGTTGAG forward; ATAGGGTACTTGGTCAGG reverse), Gapdh (TGCGACTTCAACAGCAACTC forward; ATGTAGGCCATGAGGTCCAC reverse), Gusb (GTACTCCTTGGAGGTGAA forward; TGAATCCTCGTGCTTATTG reverse). The results are presented as fold increases relative to levels observed in mock-infected control mice.

#### Western Blot Analysis

Whole lungs of female and male infected mice (*n* = 9/group) were homogenized in RIPA lysis buffer [20 mM Tris–HCl pH 7.5, 150 mM NaCl, 1 mM Na_2_EDTA, 1 mM EGDA, 1% NP-40, 1% sodium deoxycholate, 2.5 mM sodium pyrophosphate, 1 mM β-glycerophosphate, 1% Triton X-100, and 0.1% sodium dodecyl sulfate (SDS)] supplemented with phenylmethylsulfonyl fluoride, protease inhibitor mixture, and phosphatase inhibitor (Sigma-Aldrich, Milan, Italy). Lung lysates were incubated for 30 min on ice and then centrifuged at 13,000 × *g* for 30 min. The protein concentration of the supernatants was determined with the Micro BCA Protein Assay Kit (Pierce, Monza, Italy). Samples were separated by SDS-PAGE, blotted onto nitrocellulose membranes, blocked with 10% non-fat dry milk, and stained with primary (see below) and secondary antibodies peroxidase-conjugated (Jackson ImmunoResearch, Milan, Italy). Blots were developed with the ECL-Plus Detection System (GE Healthcare, Milan, Italy) and subjected to densitometry with the Quantity One Program (Bio-Rad, Milan, Italy).

Primary antibodies included rabbit polyclonal anti-NOX4, anti-phospho-p38, anti-Bcl-2 (Santa Cruz Biotechnology, Dallas, TX, USA); rabbit polyclonal anti-PRDX1 (Abcam); and mouse monoclonal anti-actin (Sigma-Aldrich).

### Statistical Analyses

The long-rank test was used to assess the difference in the overall Kaplan–Meier survival curves. Variations on bw and temperature were assigned by using a linear mixed model for repeated measures adjusted by baseline value followed by *post hoc* analysis (Bonferroni’s correction). The Wilcoxon test was performed to compare the values of Glutathione, viral M1 RNA copies, and protein concentrations in BALF in the two groups (all statistical analyses were performed using R version 3.3).

Unpaired two-tailed Student’s *t*-test or one-way ANOVA test were used for antioxidant enzyme activity; gene and protein expression; cytokine levels; alveolar area in both sexes (statistical analysis was performed using GraphPad Prism™ software version 6.0).

*p*-Values of less than 0.05 (*p* ≤ 0.05) were considered statistically significant.

## Results

### Female Mice Are More Resistant to Influenza Virus Infection Than Males

**Figure 1:**
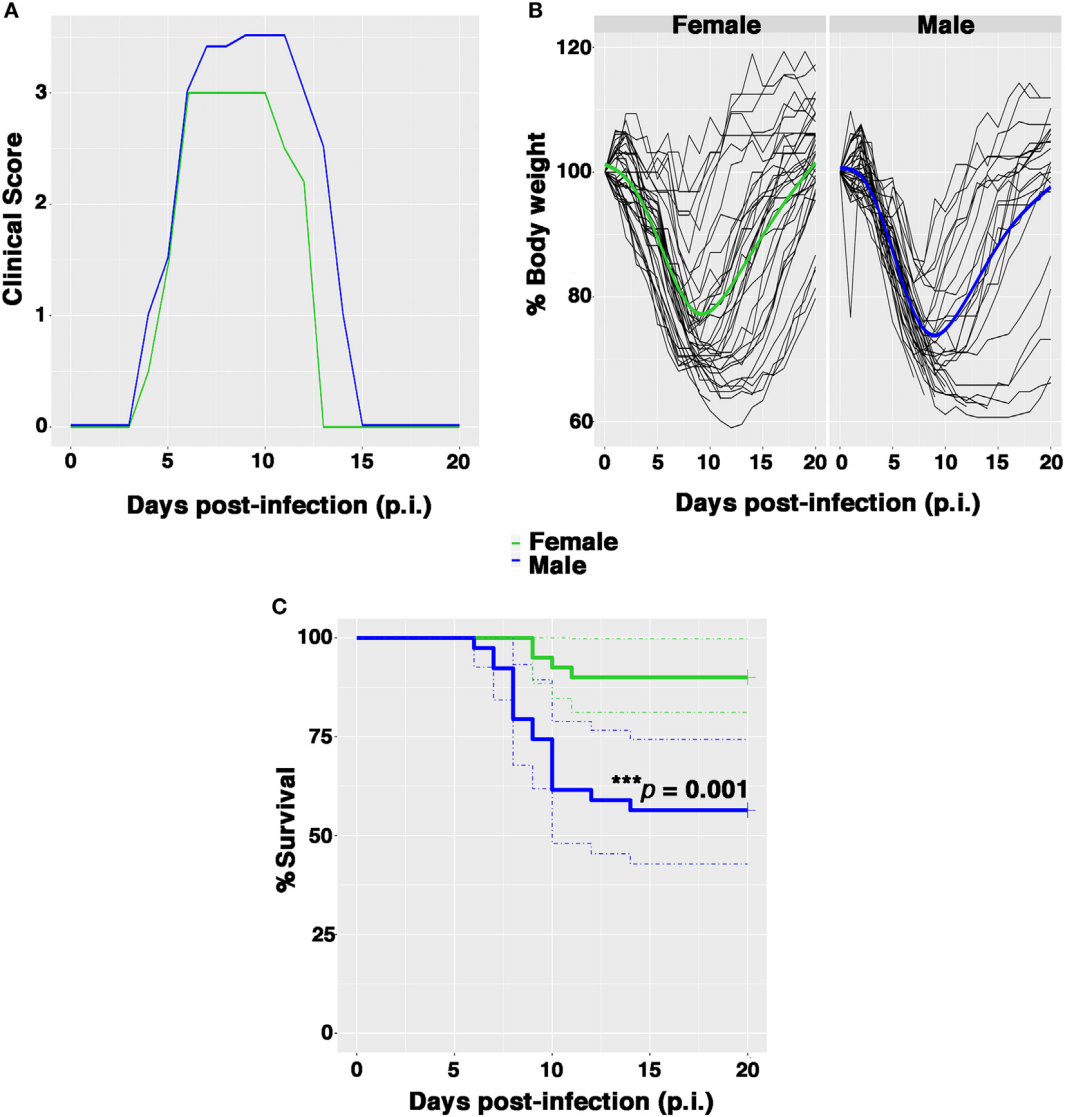
Female mice are more resistant than males to PR8 infection. Female and male mice were monitored for 21 days after intranasal inoculation with 0.5 plaque-forming unit of mouse-adapted influenza A virus (PR8). **(A)** Clinical scores: the graph represents the combined results of two separate experiments, each performed with 10 male and 10 female animals. Scores ranged from 0 (no disease) to 4 (signs and symptoms that are indicative of pneumonia). See Section “Materials and Methods” for details. **(B)** Spaghetti plot of the daily body weight (expressed as percentage respect to day 0); the bold lines represent the overall trend. **(C)** Kaplan–Meier overall survival curves. Results represent data pooled from four independent experiments, each performed with 10 males and 10 females (*n* = 40/sex), ***p-value = 0.001.

Female and male Balb/c mice were infected intranasally with 0.5 PFU/mouse and clinical signs of infection, bw, body temperature, and survival were monitored daily until 21 days after infection. The clinical responses and survival rates observed in female and male mice up to 21 days p.i are shown in Figure 1. The first symptoms of disease (piloerection, reduced food intake, and lethargy) appeared in both sexes 4 days p.i. and increased rapidly in intensity. The males exhibited more pronounced horripilation (as the first sign of pain) than the females and higher clinical scores at peak disease intensity (on p.i. days 6–12) (Figure 1A). In addition, the percentage of bw decreased rapidly in each male, whereas for some females the bw did not decrease considerably. The overall trend of bw loss at day 9 p.i. was higher in males than in females (26.6 and 23.2%, respectively), even if no statistically substantial differences were detected (Figure 1B; Figure S1A in Supplementary Material). In terms of body temperature, no differences were observed in the two groups as well (Figure S1B in Supplementary Material).

Nevertheless, the percentage of survival following infection was significantly lower among males in comparison to females (53.8 and 90%, respectively, log rank ***p-value = 0.001) (Figure 1C). Furthermore, the average day of death occurred earlier in male than in female group (on p.i. day 7 vs. on p.i. day 10).

### Influenza Virus Causes More Severe Lung Damage in Male Mice

**Figure 2:**
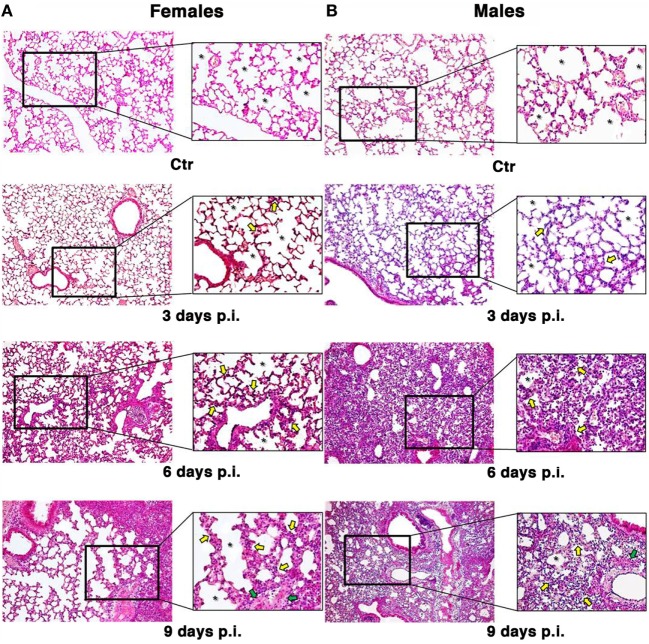
Influenza A virus produces more severe lung damage in male mice. Hematoxylin & eosin-stained section of the female **(A)** and male **(B)** pulmonary tissues from mock-infected (Ctr) or infected mice with influenza A virus and sacrificed at different times (3, 6, and 9 days). Early structural changes caused by influenza virus in the epithelium of the lower airway are variable, with cytonecrosis involving shrinkage, decreasing in alveolar surface, followed by desquamation of these cells into the luminal space. In addition, there is necrosis of the bronchiolar wall, with submucosal edema and vascular congestion. These structural changes are irregularly distributed among male and female mice. In fact, the female mice **(A)** sacrificed after 3 days still show an higher amount of alveolar surface (see the asterisks) with some initial alterations, such as thickening of the alveolar septa and inflammatory infiltration (see yellow arrows) compared to the control and the corresponding male samples **(B)**. After 6 days, we found an increase in inflammation both in male and female mice, the epithelial layer is desquamating, and necrotic epithelial cells are present in the lumen (see green arrows). But, in male **(B)**, massive pulmonary edema and hemorrhage with the alveolar air spaces fill of edema fluid and erythrocytes are also present. After 9 days, the male tissue presents a slight worsening of the previous features, whereas the female lungs start to display the same aspects, maintaining a greater alveolar area (original magnification 10×).

To look at the damage caused by PR8 infection in the lungs of mice, the animals were euthanized and lungs fixed in 10% buffered formalin prior to sectioning at 3 µm and staining with hematoxylin & eosin-stained (H&E) and Masson’s trichrome as described in Section “Materials and Methods” (Figure 2).

**Figure 3:**
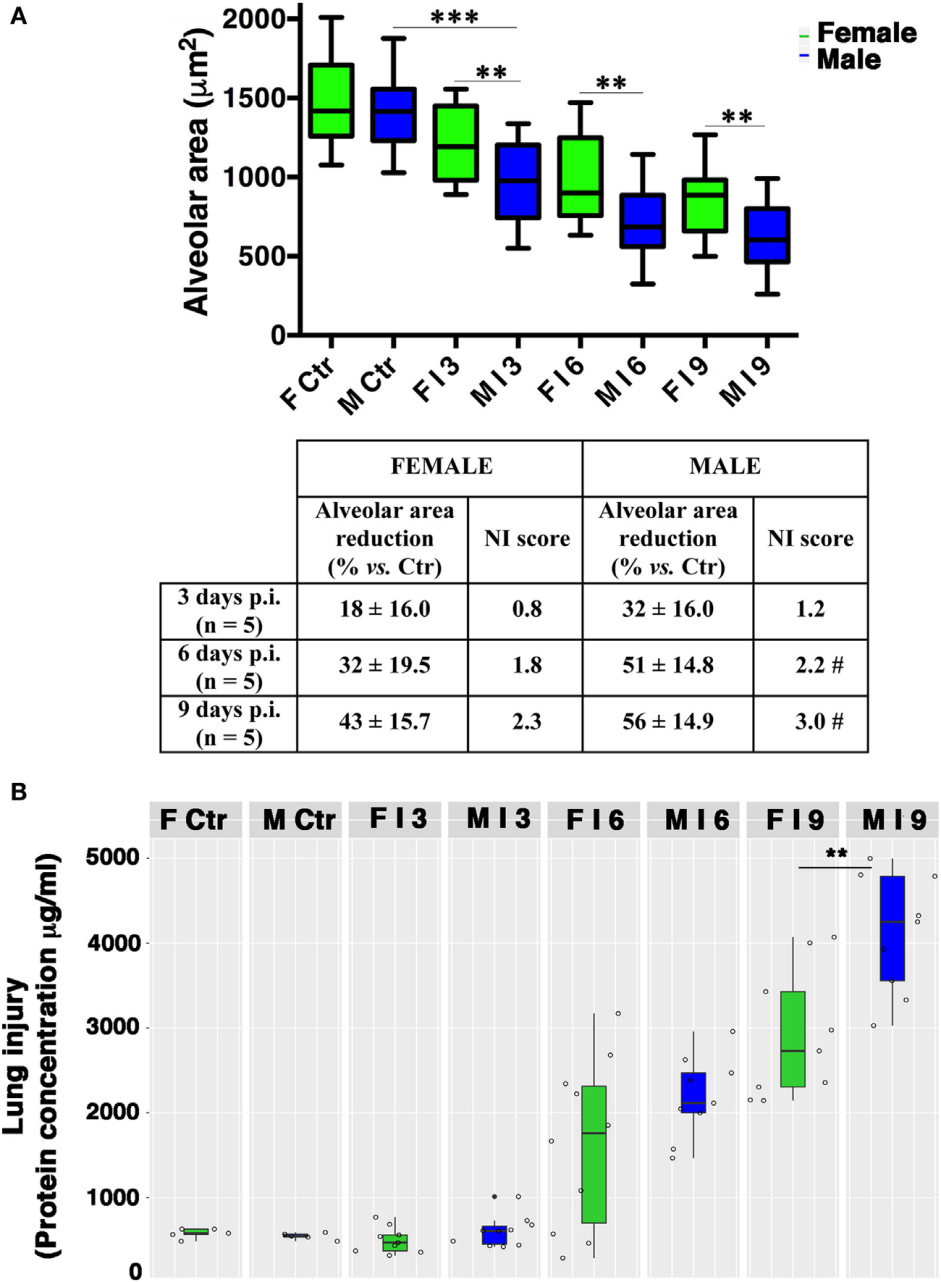
Male mice display reduced alveolar area compared with females. **(A)** Morphometric analysis at different days post-infection in female (F) and male (M) mice infected as described in Figure 2. The graph shows box-plots of alveolar area (μm^2^) of PR8-infected (I) and mock-infected mice (Ctr). **p-Value <0.01 females vs. males (unpaired *t*-test); ***p-value <0.001 Infected vs. Ctr (One-way ANOVA Bonferroni multiple comparisons test). On table below the graph, the percentage of reduction of free to air exchange vs. mock-infected (considered 100%), and the score of inflammation and necrosis (NI score) are shown. **^#^**Relevant reduction of the alveolar area, thickening of the alveolar septa, vascular congestion. **(B)** Box-plots of protein concentrations in the BALF from mock-infected (Ctr) and PR8-infected female and male mice at the time points indicated. Results represent data pooled from three separate experiments. In details, mock-infected mice were 10 (5/sex), infected mice on p.i. day 3 were 19 (9 females and 10 males), on p.i. day 6 were 19 (10 females and 9 males), on p.i. day 9 were 18 (9/sex), **p-value = 0.006.

The observation of lung tissues from uninfected control mice (*n* = 5/sex) did not highlight lesions in any of the sacrificed animals. No changes were found in the normal architecture of pulmonary parenchyma, as well as in the normal morphology of airways, alveolus, and alveolar septa. The free alveolar area for these animals (Ctr) compared with that measured in infected mice (I) is reported in Figures 2 and 3A. Results are shown for 3, 6, and 9 days p.i., since on day 21 p.i. both female and male mice that survived did not show significant differences. As reported on the table under the graph (Figure 3A), the percentage of reduction of free alveolar area in males was higher than in females.

In details, in PR8-infected male mice (*n* = 5) sacrificed after 3 days p.i. (Figures 2B and 3A), we described initial alterations of lungs parenchyma; alveolar area resulted slightly reduced compared with control lungs with thickened alveolar septa. In addition, all samples showed peripheral edema and alteration of epithelium with inflammatory cells adhering to the surface of bronchioles. The mean ± SD of free alveolar area was 975 ± 235 with a % reduction respect to Ctr of 32 ± 16. Female mice lungs (*n* = 5) at 3 days p.i. (Figures 2A and 3A) displayed a similar histopathological damage and the mean ± SD of alveolar area was 1,204 ± 238 with a % reduction of 18 ± 16.

In infected male mice (*n* = 5) sacrificed after 6 days p.i. (Figures 2B and 3A), we found widespread impairment of pulmonary parenchyma; the pictures of interstitial pneumonia were characterized by the presence of higher inflammatory exudate (interstitial and alveolar) with inflammatory cells, fibrin, cellular debris, and obvious vascular congestion and areas of necrosis. The alveolar area is greatly decreased if compared with the control lungs and strikingly, the lungs of the male mice displayed signs of more severe damage than those of the females consisting of bronchiolitis, peri-bronchiolitis, interstitial edema, alveolar wall thickening, dense interstitial granulocyte, and lymphocyte infiltrates, and the alveolar area was 708 ± 212 with a % reduction of 51 ± 14.8. By p.i. day 6, these lesions already involved over 25% of the considered parenchyma, and similar involvement was observed in survivors sacrificed 9 days p.i. Female mice lungs (*n* = 5) at 6 days p.i. (Figures 2A and 3A) showed similar histopathological alterations from a qualitative point of view, but larger preserved parenchymal areas; therefore, the alveolar area was significantly higher than male mice and this difference persisted for the duration of the experiment, indicating that the virally induced inflammation had a lower impact on lung’s female (alveolar area: mean ± SD 996 ± 286 with a % reduction of 32 ± 19.5).

In infected male mice lungs (*n* = 5) at 9 days p.i. (Figures 2B and 3A), diffuse impairment of pulmonary parenchyma was observed. The “alveolar area” was greatly reduced compared with the lung of the controls but lightly reduced compared with the animals after 6 days p.i. (free alveolar area 625 ± 213 with a % reduction of 56 ± 14.9). Mice female lungs (*n* = 5) at 9 days p.i. (Figures 2A and 3A) presented chronic flogistic infiltrate with prevalent interstitial localization activated by epithelial/endothelial lesions: the picture is similar to that of male animals, but there are a lower incidence of collagen and exudative deposition and necrosis; moreover, reconstitution areas of the alveolar epithelium is observed in female lungs (free alveolar area 837 ± 230 with a % reduction of 43 ± 15.7).

Finally, as an indirect measure of the diffuse alveolar damage, protein concentrations in BALF samples from PR8-infected mice and mock-infected controls (Ctr) were assessed. As shown in Figure 3B, increases were observed on p.i. day 6 in infected animals of both sexes. By p.i. day 9 (when maximal lung damage was noted in lung sections), BALF protein levels were significantly higher in the male group (**p-value = 0.006).

**Figure 4:**
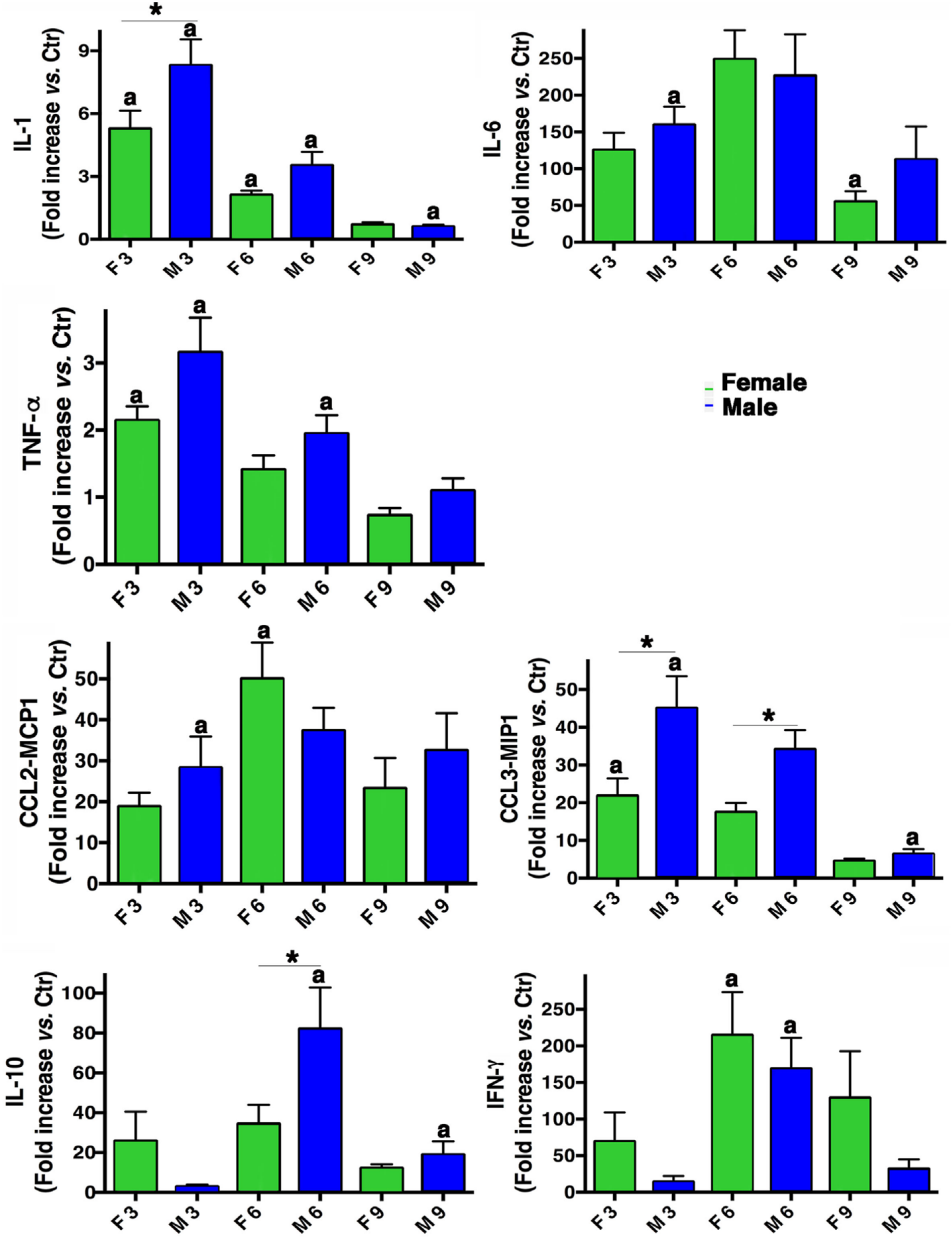
Inflammatory cytokine production in infected mice. BALF concentrations of IL-1, IL-6, TNF-α, IL-10, IFN-γ, CCL2-MCP1, and CCL3-MIP1 were measured in male (M) and female (F) mice from the mock-infected (Ctr) and PR8-infected groups. The data (mean ± SEM) are represented as the concentration of cytokines at days 3, 6, and 9 p.i. relative to Ctr. Results are obtained from five different experiments, each performed with seven male and seven female mice. *p-Value <0.05 (female vs. male group); ^a^*p*-value <0.05 (differences within a sex across time-points p.i.).

From a molecular point of view, a panel of different inflammatory cytokines and chemokines (IL-1, IL-6, TNF-α, IL-10, IFN-γ, CCL2-MCP1, and CCL3-MIP1) was evaluated in BALF from males and females mice. As shown in Figure 4, both sexes produced all the cytokines and their levels were higher than those measured in mock-infected mice. In fact, the pro-inflammatory cytokines IL-6, TNF-α, and IL-1 were increased in both sexes, the latter particularly in males. Instead, IFN-γ cytokine levels resulted more pronounced in females. Regarding chemokines CCL2-MCP1 and CCL3-MIP1, an increase was observed in both sexes, with MIP-1 higher in males. The immunosuppressive cytokine (IL-10) was increased in males on day 6 p.i.

These results apparently contradicted most of the literature that report that adult female mice experienced a greater morbidity and mortality after influenza virus infection than males, and this was correlated to immunopathology (24, 49); because hormones affect the immune response to viral infection, we wondered what are the hormonal levels in our model. We found plasma estradiol levels of 39.75 ± 18.6 pg/ml in control female mice and 30.3 ± 9.5 pg/ml in infected females (p.i. day 6); regarding testosterone, we measured 18.74 ± 6.2 ng/ml in control males and 15.12 ± 1.6 ng/ml in infected males (p.i. day 6); therefore, no significant differences in hormone level between uninfected and infected mice were detectable.

**Figure 5:**
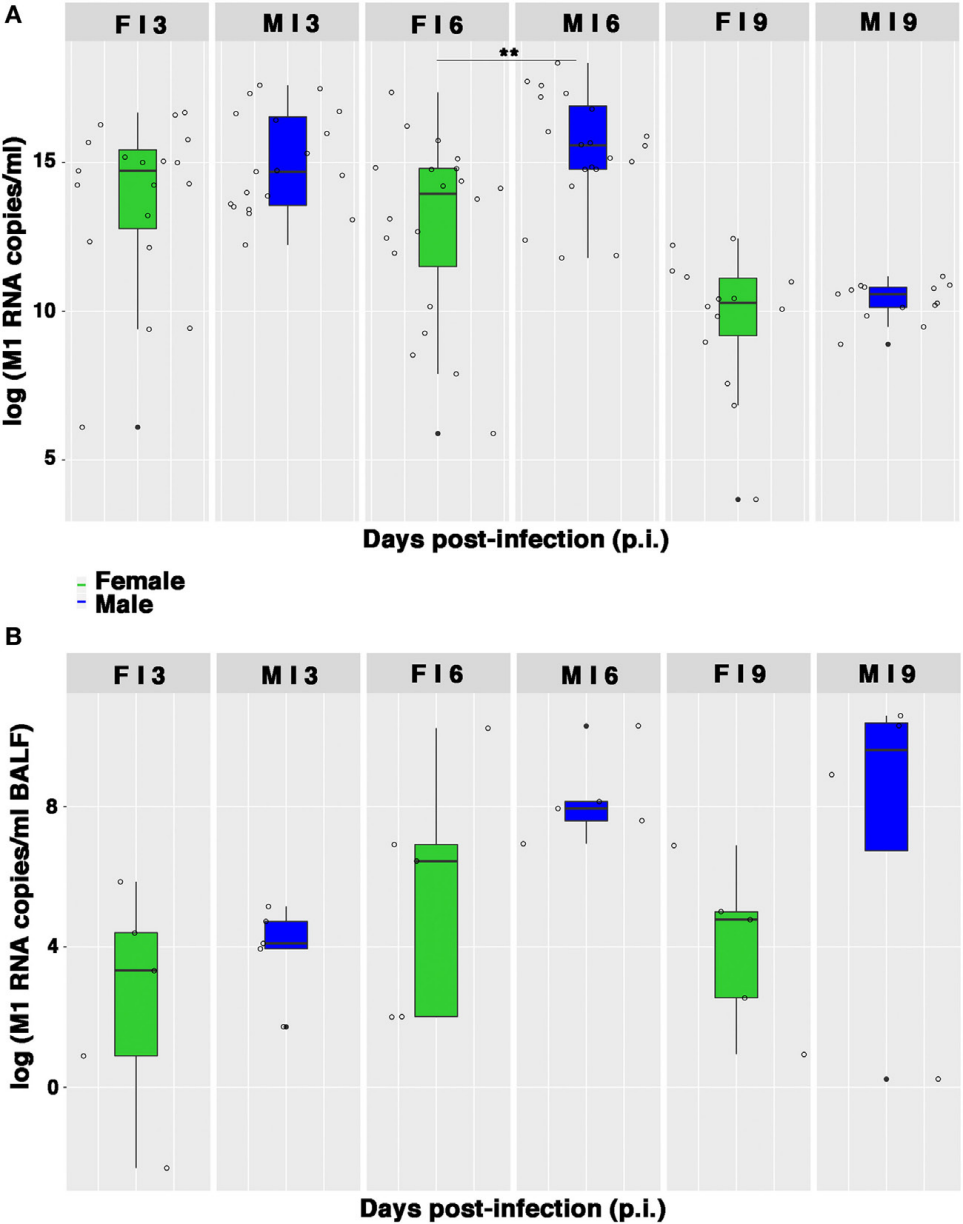
Viral M1 RNA copies are highly produced by infected males. **(A)** Box-plots of the viral M1 RNA copies measured by quantitative RT-PCR in homogenates of lungs collected on p.i. days 3, 6, and 9. Results represent data pooled from four independent experiments, each performed with five females and five males for each time-point (*n* = 20/sex), **p-value = 0.004. **(B)** Box-plots of the viral M1 RNA copies in BALF measured by RT-PCR on p.i. days 3, 6, and 9. Data shown are from one of the three experiments performed (each with five male and five female mice).

As the lung damage appeared less severe in females and on the basis of the results from hormone quantification, which seemed not to change during infection, we finally looked at the viral replication. As displayed in Figure 5A, viral M1 RNA copies in lung homogenates were consistently higher in the male group, and this difference was statistically significant on p.i. day 6 (**p-value = 0.004). Similarly, viral M1 RNA copies measured in BALF samples were also considerably higher in males than in females, during peak illness (Figure 5B). Accordingly, on p.i. day 6, the TCID_50_ measured on lung homogenates obtained from infected male mice was higher than in female mice (1,582 ± 457 and 654 ± 32 U/ml, respectively).

Therefore, collectively these data suggest that the higher morbidity and, consequently, the lower survival, as well as the more severe and extended lung damage exhibited by PR8-infected male mice, may be the result of a higher replication of influenza virus in the lungs of the male mice.

### Enhanced Systemic Antioxidant Power Protects Female Mice During Viral Infection

**Figure 6:**
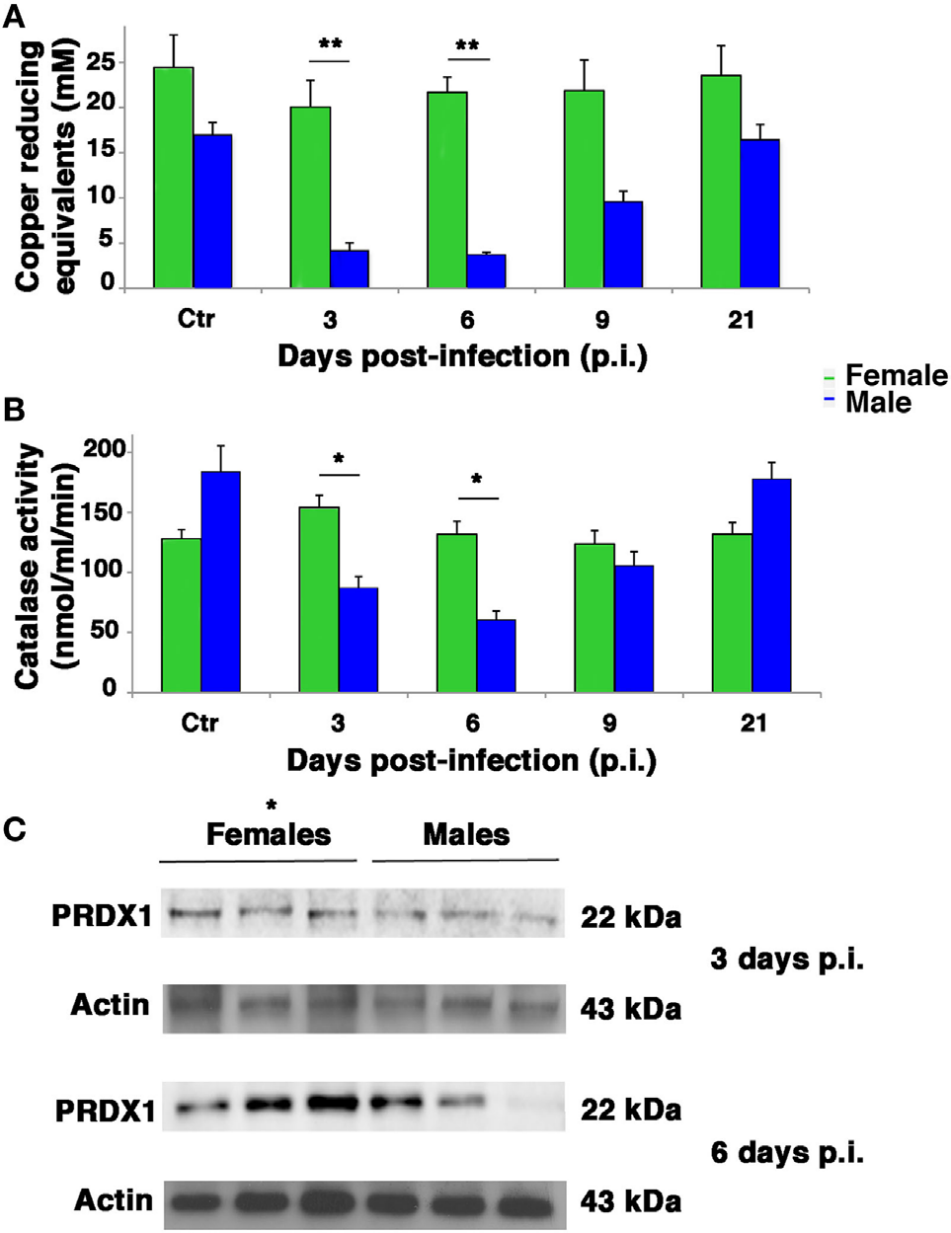
Antioxidant defenses are higher in infected female mice. **(A)** Serum from mock-infected (Ctr) and PR8-infected mice was collected on p.i. days 3, 6, 9, and 21 and assayed with the potential antioxidant test, which measures the total antioxidant power in terms of the sample’s ability to reduce copper. **(B)** Lung homogenates from Ctr and PR8-infected mice were assayed for CAT activity at the same time points than in panel **(A)**. Each value reported represents the mean ± SD of results from two separate experiments, each performed in duplicate (*n* = 4), *p-value <0.05 and **p-value <0.01. **(C)** Peroxiredoxin (PRDX)1 protein expression was analyzed by western blotting in the lungs of infected female and male mice. Actin was used as loading control. Blot shows three animals for each sex (*p-value ≤0.05) and is representative of three independent experiments performed.

Influenza virus infection is known to be strongly conditioned by host redox environment, including the intracellular GSH content, antioxidant defense, and expression of redox-regulated cell pathways (2, 9–13, 17, 18). To determine whether these factors contributed to the sexual disparities in susceptibility to PR8 infection observed in our model, we first compared the TAC of female and male mice. The TAC reflects the abundance of antioxidant molecules and enzymes available in the blood to counteract the effects of ROS/reactive nitrogen species, such as those produced during viral infection. As shown in Figure 6A, mock-infected female mice displayed appreciably higher TAC than their male counterparts. More striking sex-related differences were seen in PR8-infected mice. The reduction potential of serum from female mice remained high (near baseline levels) throughout the viral infection, whereas that of the males dropped significantly. On p.i. days 3 and 6, the TAC recorded for the males was significantly lower than those of the females (unpaired *t*-test **p-value <0.01). By p.i. day 21, TAC of surviving animals had returned to their respective baseline levels, which were once again lower in males. Accordingly, the analysis of free thiols in serum and lung homogenates from infected and mock-infected mice showed a slight reduction in infected males compared with mock infected, while no differences were detectable between infected and non-infected females (data not shown).

Next, we assessed antioxidant enzyme activities in lung homogenates. As shown in Figure 6B in mock-infected controls, CAT activity did not significantly differ among males and females. After PR8 infection, however, CAT activity in the lungs of male mice dropped substantially, reaching levels on p.i. days 3 and 6 that were significantly lower than those of the female group, which remained stable throughout the viral infection (unpaired *t*-test *p-value <0.05).

Activity of SOD increased appreciably in both sexes on p.i. day 3, but this change was not statistically significant. Essentially, PR8 infection was not associated with any significant change in pulmonary SOD activity in either the female or male mice, and no significant sex-related differences were observed at any of the time points (Figure S2 in Supplementary Material). Furthermore, we measured GSHPx activity, finding that it decreased significantly in both sexes but in greater extent in infected male mice than female. To note that female mock-infected mice showed significantly higher basal activity of this enzyme (Figure S3 in Supplementary Material). Finally, the expression of another antioxidant enzyme, peroxiredoxin (PRDX)1, was analyzed by western blot in lung of infected female and male mice at p.i. days 3 and 6 (time when the maximal difference in redox conditions was observed). As shown in Figure 6C, the expression of this enzyme was higher in females than in males. The densitometric analysis of ratio PRDX1/actin of three animals for each sex at 3 and 6 days p.i. was 1.5- and 3-fold higher, respectively, unpaired *t*-test *p-value ≤0.05, suggesting that females are more protected by influenza for the presence of reducing conditions.

### The Intracellular Content and Biosynthesis of GSH Are Preserved in Infected Female Mice

**Figure 7:**
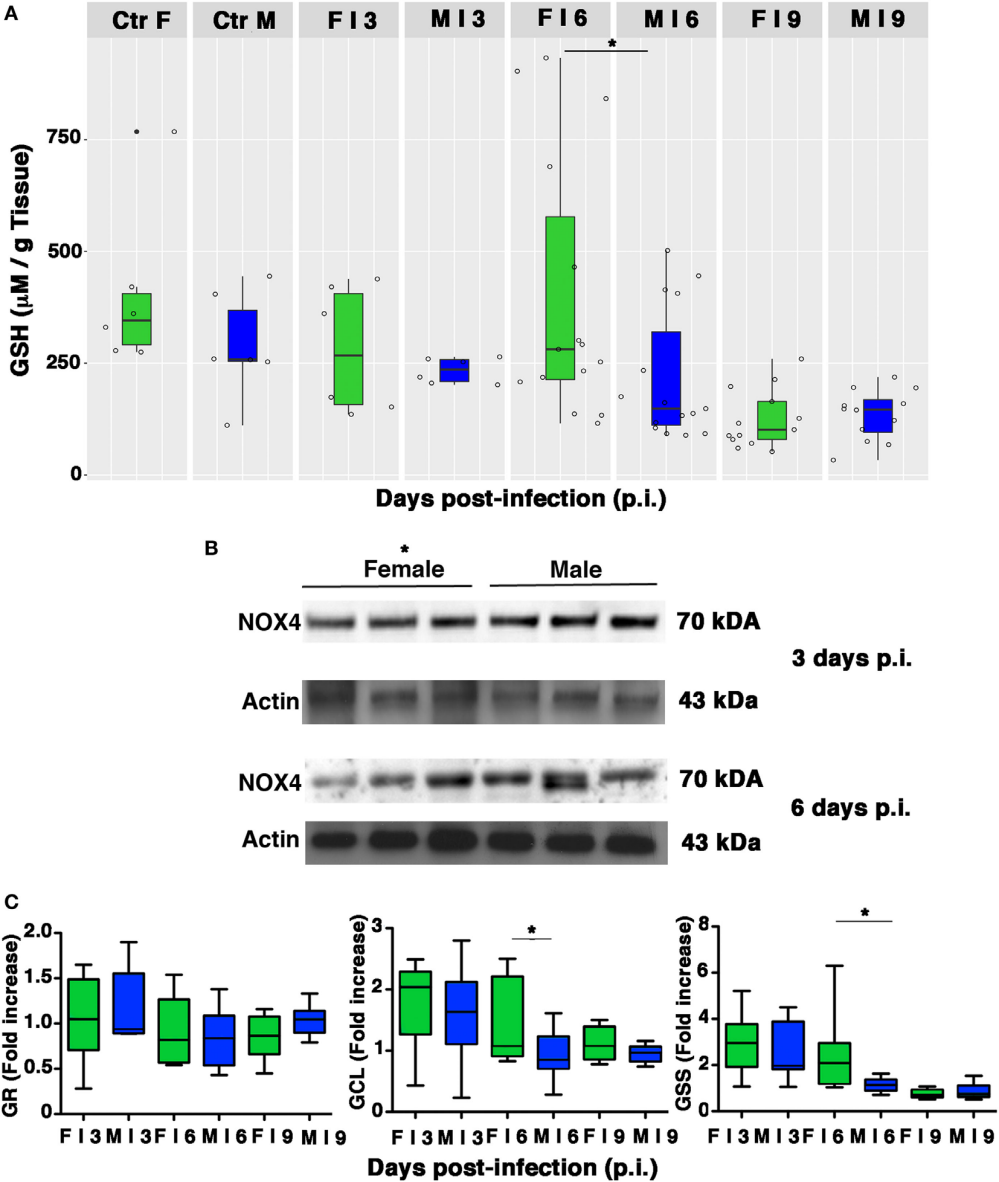
Pulmonary intracellular reduced glutathione (GSH) levels are higher in females than in males. **(A)** GSH content was measured in the lungs of mock-infected (Ctr) or infected (I) female and male mice sacrificed at indicated time points. Results represent data pooled from four separate experiments. In details, mock-infected mice were 12 (6/sex), infected mice on p.i. day 3 were 12 (6/sex), on p.i. day 6 were 30 (15/sex), on p.i. day 9 were 25 (13 females and 12 males) *p-value = 0.034. **(B)** NADPH oxidase 4 (NOX4) expression was analyzed by western blotting in the lungs of infected female and male mice. Actin was used as loading control. Blots shown are one representative experiment of three performed (three animals for each sex, *p-value ≤0.05). **(C)** RT-PCR quantification of enzymes responsible for recycling and biosynthesis of GSH [glutathione reductase (GR), glutamate cysteine ligase (GCL), and glutathione synthase (GSS)]. Gene expression was measured in lung homogenates of animals sacrificed on p.i. days 3, 6, and 9. Box-plots represent the fold increases relative to levels observed in mock-infected controls (*n* = 8/sex). Unpaired *t*-test *p-value ≤0.05.

Influenza virus infection is associated with reductions in the GSH content of infected cells, which facilitate viral replication (2, 9–12). As shown in Figure 7A, basal levels of GSH in lung homogenates from the mock-infected control group were slightly higher and less variable in females than in males. As expected, levels decreased in both sexes after infection, but on p.i. day 6, there was a sharp drop in the GSH content of male lungs, which resulted in significantly lower levels than those found in females (*p-value = 0.034). Interestingly, this drop coincided with the time of viral loads peak in the lungs of the male mice.

Reduced glutathione depletion may be due to its buffering role against ROS that, during viral infection, essentially derive from NOX4 (11). Therefore, we evaluated the expression of this enzyme in the lung homogenate of females and males. Densitometric analysis of three animals for both sexes demonstrated that NOX4 was less expressed in females than in males (3 and 6 days p.i., 1.5-fold lower, unpaired *t*-test *p-value ≤0.05; Figure 7B).

Intracellular GSH is regenerated from the oxidized form (GSSG) by GR or synthesized *ex novo* by the consecutive actions of GCL and GSS (50). Our next step was thus aimed at determining whether the sex-related differences in pulmonary GSH levels were also associated with differences in transcriptional expression of these three enzymes. As shown in Figure 7C, compared with their male counterparts, female PR8-infected mice showed a greater upregulation of GCL and GSS expression, suggesting more efficiency in counteracting PR8-induced GSH depletion (unpaired *t*-test *p-value <0.05). Collectively, these results indicate that female mice have an intrinsically higher antioxidant capacity, and during PR8 infection they are also capable of more efficient restoration of the physiological redox milieu in terms of GSH content that could be due to the upregulation of its synthesis.

### Lung Homogenates From Females Contain Higher Levels of the Anti-Apoptotic Bcl-2 Protein

**Figure 8:**
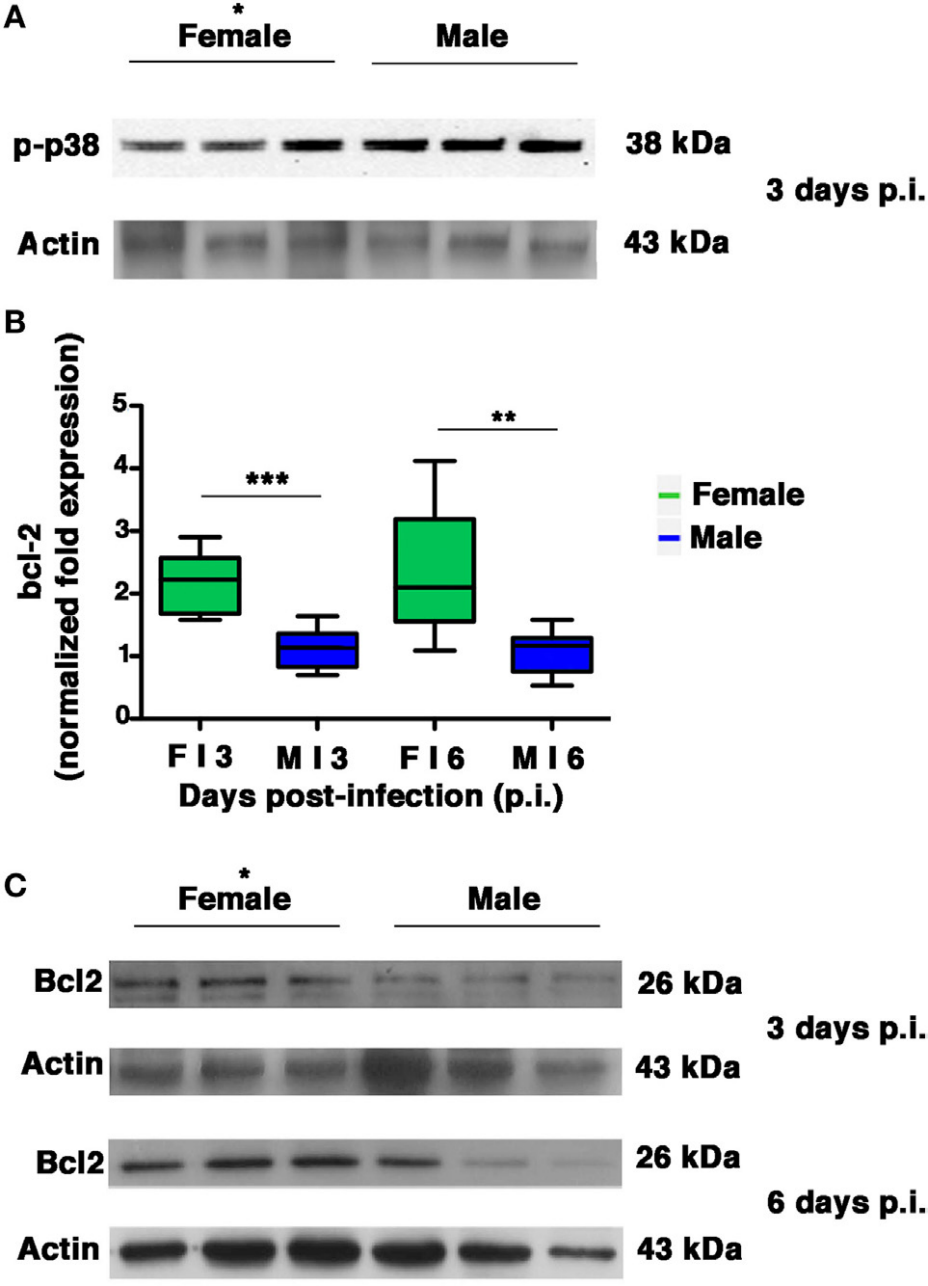
Female mice respond to PR8 infection with marked upregulation of Bcl-2 expression. **(A)** p-p38MAPK expression was analyzed by western blotting in the lungs of infected female and male mice at 3 days p.i. (three animals for each sex, *p-value = 0.02). **(B)** RT-PCR quantification of *bcl-2* gene expression in the lung homogenates of infected mice euthanized on p.i. days 3 and 6. Box-plots represent the fold increases relative to levels observed in mock-infected controls (*n* = 8/sex), unpaired *t*-test **p-value = 0.0018; ***p-value = 0.0002. **(C)** Bcl-2 protein expression was analyzed by western blotting in the lungs of infected female and male mice. Actin was used as loading control. Blots shown are one representative experiment of three performed (three animals for each sex, *p-value ≤0.05).

Several intracellular redox-regulated pathways are involved in regulation of influenza virus replication, particularly the kinase p38MAP that is activated by NOX4-derived ROS (11). In cells that are highly permissive to viral infection, activated p38MAPK is entirely addressed to the nucleus, in which it participates efficiently in vRNP phosphorylation. In cells that are characterized by high levels of GSH and abundant expression of the anti-apoptotic protein Bcl-2, influenza virus replication is reduced (2). The inhibition is due to co-localization of activated p38MAPK with its cytosolic substrate (Bcl-2) and block of its translocation to the nucleus. As a consequence, NP is retained in the nucleus and viral replication is inhibited (18). Thus, we decided to evaluate whether the differences in viral load observed between the two sexes were also related to differences in p38MAPK activation and in Bcl-2 expression in the lungs. We found that p38MAPK was early activated in both groups on p.i. day 3 (Figure 8A). However, densitometric analysis of three different animals revealed that the kinase was more activated (almost twofold) in males compared to three homogenates of females (unpaired *t*-test *p-value = 0.02), thereby indicating more efficiency of p38MAPK in males. Afterward, we evaluated the expression of Bcl-2 (both mRNA and protein) in the lungs of females and males on p.i. days 3 and 6. We found that during viral infection, female mice exhibited more substantial *bcl-2* gene upregulation compared with males (Figure 8B unpaired *t*-test: **p-value = 0.0018; ***p-value = 0.0002). Specifically, *bcl*-2 transcript levels in females were approximately two times as high as those found from mock-infected controls. On the contrary, there was no significant upregulation in the male mice. At the same time, densitometric analysis three different animals revealed that Bcl-2 protein levels found in the lung homogenates were also clearly higher in the female group (Figure 8C, 3 and 6 days p.i., unpaired *t*-test *p-value ≤0.05).

All these results indicate that during PR8 viral infection, females activate transcriptional processes to maintain high levels of Bcl-2 protein. This event might contribute to keep p38MAPK in the cytosol and to inhibit NP traffic and viral replication.

## Discussion

In this article, we focused on one of the *in vivo* mechanisms contributing to sex-related disparities in influenza virus infection. In particular, we pointed at systemic and organ redox state as critical determinant for influenza virus replication. We found that female mice infected with PR8 displayed a higher survival rate, milder clinical disease, and lower pulmonary viral loads than their male counterparts. These sex-based disparities correlate largely on differences between the redox conditions in the female and male animals. Mock-infected female mice have an intrinsically higher antioxidant capacity, measured as total serum antioxidant power and GSH content in lung homogenates. These better physiological conditions persist during viral infection when we observed: upregulation of enzymes responsible for GSH biosynthesis, higher level of PRDX1, maintenance of CAT activity, and a less decrease of GSHPx activity. Infected females display also higher expression (at the mRNA and protein levels) of the anti-apoptotic protein Bcl-2, which is involved in the regulation of specific steps of influenza virus replication (2, 18). On the other hand, infected male mice displayed high expression of NOX4 enzyme, and increased levels of phosphorylated p38MAPK.

The impact of sex on susceptibility to viral infections has been hypothesized several years ago (51). Generally, females and males of various species respond differently to many DNA and RNA viruses. The mechanisms underpinning sex differences in response to viral infections are controversial, and roles for immunological, hormonal, behavioral, epigenetic, and genetic factors have all been proposed (20, 52).

It has been shown that females generate stronger innate and adaptive immune responses than males, with immune cells higher in number and activity, as well as with higher antibodies levels than males (21–23). This immunological advantage contributes to virus clearance, but on the other hand it makes females more prone to autoimmune diseases and to infectious disease-derived immunopathology (21–23). In fact, infectious diseases pathogenesis derives both from the pathogen and from the host immune response (22, 23). Influenza viruses can cause severe disease as interstitial pneumonia and bronchiolitis, characterized by typical inflammatory anatomical–pathological lesions and sometimes, massive hemorrhage, with interstitial, bronchiolar, and alveolar localization (53).

In addition to a massive cell infiltrates in the infected lungs, there is an overproduction of several pro-inflammatory cytokines and chemokines (54, 55). In fact, in this study, we observed histopathological alterations relative to interstitial pneumonia and in line with this observation, we found high levels of IL-1, IL-6, TNF-α, IL-10, IFN-γ, CCL2-MCP1, and CCL3-MIP1 in infected mice of both sexes.

However, the picture in female mice appeared different because of larger preserved parenchymal areas and a consequently total alveolar area significantly higher than in male mice. This difference persisted during the infection, suggesting that the virally induced inflammation had a lower impact on lung’s female. Hormones exert a complex role in inflammation, in particular, estradiol enhances inflammation at low doses, but reduces it at higher concentrations (56), while low concentration of testosterone has reported to have a negative impact on the outcome of influenza disease (57). In our model, we found basal levels of estradiol, which did not significantly change with the infection, similar to that reported by Robinson et al. (24). Although a slight decrease, testosterone as well did not significantly vary with the infection. Therefore, in the attempt to explain the less severe lung damage that we observed in females, especially between 6 and 9 days p.i., we looked at the pathogen: we found lower viral titer in lung homogenates and BALF from female mice, with a lower infectivity, as shown by TCID_50_. So, these results lead us to argue that the lower impact observed in females is related to a less extent of virus replication and spread, more than a host immune effect.

With this hypothesis our attention focused on viral replication and the possible redox-related mechanisms underlying sex disparity. Female and male mice differed remarkably in terms of their basal redox state and their ability to counteract virus-associated oxidative imbalance. Prior to inoculation, more reducing conditions were found in the female animals in terms of GSH levels in the lungs and the TAC. These differences are in line with those reported in VSMC isolated from the aortas of male and female rats (35). *In vitro* data suggest that this sexual dimorphism can be maintained after the induction of oxidative stress, which results in females displaying greater resistance to oxidative injury and an increased capacity to counteract it (35). For example, some authors report different sex-dependent susceptibility to cytotoxic agents and treatments that is related to the incapacity of XY neurons to maintain GSH intracellular levels (58). Our *in vivo* findings support this view: during the course of influenza virus infection, the intrinsic redox balance, i.e., reducing conditions, was more effectively maintained in the female mice. In these animals, inoculation was promptly followed by the activation of enzymes involved in biosynthesis of GSH aimed at counteracting the GSH depletion induced by the virus. The period of upregulated GSH synthesis and higher levels of GSH in the females coincided with the period characterized by peak viral loads in males.

Thiols are key players in conditions of oxidative stress. Most non-protein antioxidants as well as antioxidant enzymes are thiol based (59). GSH acts as radical scavenger by directly neutralizing a variety of reactive molecules, like superoxide anion and hydroxylradicals (60), and indirectly through enzymatic reactions being a cofactor of GSHPx (61). Here, we found in male mice, higher levels of NOX4, one of the major enzymes producing ROS, thus suggesting that GSH depletion in males might be due to its consumption for its ROS buffering function. In fact, we have previously demonstrated that inhibition of NOX4 activity through chemical inhibitors or RNA silencing blocks the influenza virus-induced ROS increase, restores the content of GSH, and inhibits viral replication (11). Interestingly, several studies demonstrate that estrogens inhibit ROS production (56) by modulating antioxidant enzyme activities (62); estrogen levels have been shown also to be positively correlated to GSHPx activity in women, while no significant correlation was observed with SOD (63, 64) that in our model did not change between sexes. Moreover, estradiol has been shown to increase expression of GCL (65), that is the rate-limiting enzyme for the synthesis of GSH (60) and therefore, together with GSS and GSHPx, closely linked to the GSH levels. On the contrary, testosterone has been shown to have pro-oxidant effect (66, 67) and so we cannot exclude that it could contribute to viral replication in males by activating redox-sensitive pathways. We also found a drop in CAT activity in infected males, especially when the viral replication peaked at 6 days p.i. Accordingly, a time-dependent decrease in CAT activity has been observed in parallel to increase in influenza NS1-protein expression (13).

Several authors report that by restoring reducing conditions, viral replication and virus-induced host damage are inhibited, suggesting antioxidant therapy as a potential antiviral strategy (8, 9, 68–70). Indeed, various synthesized or natural compounds characterized by antioxidant activity have been proposed as anti-influenza agents (17, 71–76). For example, our group has shown that GSH treatment strongly inhibits viral replication by impairing glycoprotein folding (10); on the other hand, we have recently shown that GSH depletion increased influenza virus replication by preventing activation of innate antiviral response (7). Indeed, the role of GSH in modulating immune response is well known (8, 77–79). For example, in antigen-presenting cells, GSH depletion correlates with defective antigen processing and reduced secretion of T helper 1 (Th1) cytokines, thus favoring polarization from the typical Th1 profile toward a Th2 response (8). Furthermore, in T lymphocytes, intracellular GSH content is critical for their proliferation as well as extracellular thiols for their activation and function. Angelini et al. (80) demonstrated that exogenous thiols, i.e., free cysteine and thioredoxin, were released by monocyte-derived human dendritic cells (DCs) in the extracellular space to provide a reducing microenvironment required for T lymphocyte activation and an efficient immune response. In our study, we found a slight decrease of free thiols in the lung homogenates and serum of infected males, while no changes were observed in infected females compared with control. It would be interesting to investigate whether the observed decrement in males is due to a dysfunction of DCs and to impairment in T lymphocyte activation.

The imbalance in the redox state is fundamental for the activation of many cell factors, involved in the regulation of host response and in the control of influenza virus life cycle (16). Among them, MAPKs and Bcl-2 protein regulate the intracellular trafficking of the viral NP (18). In this study, we found that phosphorylation of p38MAPK is highly expressed in lung homogenate of males, suggesting that this phenomenon could explain in part the high viral load measured in males. Conversely, we found Bcl-2 to be highly expressed in the lungs of infected female mice, at both transcriptional and translational level. Furthermore, over-expression of Bcl-2 protein has been hypothesized to be associated with increased GSH levels (81, 82), and these characteristics have been found in lung homogenates of female infected mice. Based on this evidence, we can hypothesize that in females the more resistance to oxidative damage during PR8 infection may impair virus replication probably by blocking viral protein maturation and vRNP complex formation.

A final point to be considered in this scenario concerns the hypothesized role of autophagy, a cytoprotective host process that is subverted by the influenza virus to ensure its own replication (83). Metabolic stress appears to bolster a stronger, more sustained autophagic response in cells from females than in those collected from males (84). Therefore, we cannot exclude the possibility that more effective autophagic cytoprotection in lung cells from female mice led to a “weaker” cytopathological cascade.

In conclusion, our data suggest that the mechanisms underlying the sexual disparities observed in the host response to influenza can be ascribed in part to differences in their capacity to maintain redox homeostasis. In fact, in our model, we have found that females are more resistant to the influenza virus due to their ability to maintain reduced conditions during infection, thereby hindering completion of the virus life cycle and inhibiting viral replication. Therefore, although further studies are needed to fine characterize redox mechanisms underlying sex disparities in infections, i.e., the use of different antioxidants like *N*-acetylcysteine, GSH, or natural polyphenols, as well as the silencing of antioxidant enzymes that regulate viral replication, our findings may contribute to the identification of new targets for sex-based antiviral therapies. Indeed, generally, sex-related differences are not considered in current strategies for the prevention, management, and treatment of many diseases (85). Instead, a more detailed knowledge of the metabolic conditions that characterize the two sexes could ultimately improve our ability to provide patients with individualized therapies and cost-effective solutions.

## Ethics Statement

In accordance with national law, the experiments described in this manuscript were approved by the Italian Ministry of Health, which verified the ethical and scientific appropriateness of the research. All animals received humane treatment, and every effort was made to minimize their suffering.

## Author Contributions

IC, PChecconi, DA, MA, PColuccio, RD, and PM performed experiments. DF, AC, MT, RM, CM, AV, WM, and LN analyzed data. IC, PChecconi, EG, ATP, and LN designed and supervised experiments. IC, PChecconi, ATP, and LN wrote the paper.

## Conflict of Interest Statement

The authors declare that the research was carried out in the absence of any personal, commercial, or financial relationships that could be construed as a potential conflict of interest.

## Supplementary Material

Figure S1 Body weight (bw) and temperature in female and male infected mice. **(A)** Spaghetti plot of the daily bw (expressed in grams); the bold lines represent the overall trend. **(B)** Spaghetti plot of the daily body temperature, the bold lines represent the overall trend. Results represent data pooled from 4 independent experiments, each performed with 10 males and 10 females (*n* = 40/sex).

Figure S2 No differences in superoxide dismutase (SOD) activity were observed in infected female and male mice. Lung homogenates from Ctr and PR8-infected mice were assayed for SOD activity on p.i. days 3, 6, 9, and 21. Each value reported represents the mean ± SD of results from two separate experiments, each performed in duplicate (*n* = 4).

Figure S3 Glutathione peroxidase (GSHPx) activity is less reduced in infected female mice. Lung homogenates from Ctr and PR8-infected mice were assayed for GSHPx activity on p.i. day 6. Each value reported represents the mean ± SD of results from 3 mice/sex, each performed in triplicate (*n* = 9). One-way ANOVA test ***p*-value <0.01; ****p*-value <0.001.

## Abbreviations

BALF, broncho-alveolar lavage fluid; CAT, catalase; Gapdh, glyceraldehyde-3-phosphate dehydrogenase; Gusb, glucuronidase beta; GCL, glutamate cysteine ligase; GR, glutathione reductase; GSS, glutathione synthase; GSHPx, glutathione peroxidase; H&E, hematoxylin & eosin-stained; MLD_50_, 50% mouse lethal dose; NOX4, NADPH oxidase 4; NP, nucleoprotein; GSSG, oxidized glutathione; PRDX, peroxiredoxin; PFU, plaque-forming unit; PDI, protein disulfide isomerase; ROS, reactive oxygen species; GSH, reduced glutathione; Rpl13a, ribosomal protein L13A; SOD, superoxide dismutase; TCID_50_, tissue culture infectious dose 50%; TAC, total antioxidant capacity; VSMC, vascular smooth muscle cells.

## References

[1] Nencioni L Sgarbanti R Amatore D Checconi P Celestino I Limongi D Intracellular redox signaling as therapeutic target for novel antiviral strategy. Curr Pharm Des (2011) 17:3898–904. 10.2174/138161211798357728 21933147

[2] Nencioni L Iuvara A Aquilano K Ciriolo MR Cozzolino F Rotilio G Influenza A virus replication is dependent on an antioxidant pathway that involves GSH and Bcl-2. FASEB J (2003) 17:758–60. 10.1096/fj.02-0508fje 12594179

[3] Checconi P Sgarbanti R Celestino I Limongi D Amatore D Iuvara A The environmental pollutant cadmium promotes influenza virus replication in MDCK cells by altering their redox state. Int J Mol Sci (2013) 14:4148–62. 10.3390/ijms14024148 23429198 PMC3588091

[4] Ehrhardt C Seyer R Hrincius ER Eierhoff T Wolff T Ludwig S. Interplay between influenza A virus and the innate immune signaling. Microbes Infect (2010) 12:81–7. 10.1016/j.micinf.2009.09.007 19782761

[5] Vlahos R Stambas J Bozinovski S Broughton BR Drummond GR Selemidis S. Inhibition of Nox2 oxidase activity ameliorates influenza A virus-induced lung inflammation. PLoS Pathog (2011) 7:e1001271. 10.1371/journal.ppat.1001271 21304882 PMC3033375

[6] Olagnier D Peri S Steel C van Montfoort N Chiang C Beljanski V Cellular oxidative stress response controls the antiviral and apoptotic programs in dengue virus-infected dendritic cells. PLoS Pathog (2014) 10:e1004566. 10.1371/journal.ppat.1004566 25521078 PMC4270780

[7] Diotallevi M Checconi P Palamara AT Celestino I Coppo L Holmgren A Glutathione fine-tunes the innate immune response toward antiviral pathways in a macrophage cell line independently of its antioxidant properties. Front Immunol (2017) 8:1239. 10.3389/fimmu.2017.01239 29033950 PMC5626850

[8] Fraternale A Brundu S Magnani M. Glutathione and glutathione derivatives in immunotherapy. Biol Chem (2017) 398:261–75. 10.1515/hsz-2016-0202 27514076

[9] Cai J Chen Y Seth S Furukawa S Compans RW Jones DP. Inhibition of influenza infection by glutathione. Free Radic Biol Med (2003) 34:928–36. 10.1016/S0891-5849(03)00023-6 12654482

[10] Sgarbanti R Nencioni L Amatore D Coluccio P Fraternale A Sale P Redox-regulation of the influenza hemagglutinin maturation process: a new cell-mediated strategy for anti-influenza therapy. Antioxid Redox Signal (2011) 15:593–606. 10.1089/ars.2010.3512 21366409

[11] Amatore D Sgarbanti R Aquilano K Baldelli S Limongi D Civitelli L Influenza virus replication in lung epithelial cells depends on redox-sensitive pathways activated by NOX4-derived ROS. Cell Microbiol (2015) 17:131–45. 10.1111/cmi.12343 25154738 PMC4311438

[12] Kumar P Khanna M Srivastava V Tyagi YK Raj HG Ravi K Effect of quercetin supplementation on lung antioxidants after experimental influenza virus infection. Exp Lung Res (2005) 5:449–59. 10.1080/019021490927088 16019982

[13] Qi X Zhang H Wang Q Wang J. The NS1 protein of avian influenza virus H9N2 induces oxidative-stress-mediated chicken oviduct epithelial cells apoptosis. J Gen Virol (2016) 97:3183–92. 10.1099/jgv.0.000625 27902334

[14] Checconi P Salzano S Bowler L Mullen L Mengozzi M Hanschmann EM Redox proteomics of the inflammatory secretome identifies a common set of redoxins and other glutathionylated proteins released in inflammation, influenza virus infection and oxidative stress. PLoS One (2015) 10:e0127086. 10.1371/journal.pone.0127086 25985305 PMC4436175

[15] Yamada Y Limmon GV Zheng D Li N Li L Yin L Major shifts in the spatio-temporal distribution of lung antioxidant enzymes during influenza pneumonia. PLoS One (2012) 7:e31494. 10.1371/journal.pone.0031494 22355371 PMC3280306

[16] Nencioni L Sgarbanti R De Chiara G Garaci E Palamara AT. Influenza virus and redox mediated cell signaling: a complex network of virus/host interaction. New Microbiol (2007) 30:367–75. 18080671

[17] Palamara AT Nencioni L Aquilano K De Chiara G Hernandez L Cozzolino F Resveratrol inhibits influenza A virus replication in vitro and in vivo. J Infect Dis (2005) 191:1719–29. 10.1086/429694 15838800

[18] Nencioni L De Chiara G Sgarbanti R Amatore D Aquilano K Marcocci ME Bcl-2 expression and p38MAPK activity in cells infected with influenza A virus: impact on virally induced apoptosis and viral replication. J Biol Chem (2009) 284:16004–15. 10.1074/jbc.M900146200 19336399 PMC2708894

[19] WHO, Department of Gender, Women and Health. Sex, Gender and Influenza. Geneva, Switzerland: WHO press (2010).

[20] Klein SL Hodgson A Robinson DP Mechanisms of sex disparities in influenza pathogenesis. J Leukoc Biol (2012) 92:67–73. 10.1189/jlb.0811427 22131346 PMC4046247

[21] Libert C Dejager L Pinheiro I. The X chromosome in immune functions: when a chromosome makes the difference. Nat Rev Immunol (2010) 10:594–604. 10.1038/nri2815 20651746

[22] Vom Steeg LG Klein SL SeXX matters in infection disease pathogenesis. PLoS Pathog (2016) 12(2):e1005374 10.1371/journal.ppat.1005374 26891052 PMC4759457

[23] Klein SL Flanagan KL. Sex differences in immune responses. Nat Rev Immunol (2016) 16:626–38. 10.1038/nri.2016.90 27546235

[24] Robinson DP Lorenzo ME Jian W Klein SL Elevated 17β-estradiol protects females from influenza A virus pathogenesis by suppressing inflammatory responses. PLoS Pathog (2011) 7:e1002149 10.1371/journal.ppat.1002149 21829352 PMC3145801

[25] Hall OJ Nachbagauer R Vermillion MS Fink AL Phuong V Krammer F Progesterone-based contraceptives reduce adaptive immune responses and protection against sequential influenza A virus infections. J Virol (2017) 91(8):e02160-16. 10.1128/JVI.02160-16 28179523 PMC5375688

[26] Crighton EJ Elliott SJ Moineddin R Kanaroglou P Upshur RE. An exploratory spatial analysis of pneumonia and influenza hospitalizations in Ontario by age and gender. Epidemiol Infect (2007) 135:253–61. 10.1017/S095026880600690X 16824252 PMC2870578

[27] Jensen-Fangel S Mohey R Johnsen SP Andersen PL Sørensen HT Ostergaard L. Gender differences in hospitalization rates for respiratory tract infections in Danish youth. J Infect Dis (2004) 36:31–6. 10.1080/00365540310017618 15000556

[28] Wang XL Yang L Chan KH Chan KP Cao PH Lau EH Age and sex differences in rates of influenza-associated hospitalizations in Hong Kong. Am J Epidemiol (2015) 182:335–44. 10.1093/aje/kwv068 26219977

[29] Bonmarin I Belchior E Bergounioux J Brun-Buisson C Mégarbane B Chappert JL Intensive care unit surveillance of influenza infection in France: the 2009/10 pandemic and the three subsequent seasons. Euro Surveill (2015) 20:46. 10.2807/1560-7917 26607262

[30] Kawado M Hashimoto S Murakami Y Izumida M Ohta A Tada Y Annual and weekly incidence rates of influenza and pediatric diseases estimated from infectious disease surveillance data in Japan, 2002–2005. J Epidemiol (2007) 17(Suppl):S32–41. 10.2188/jea.17.S32 18239340 PMC4809252

[31] Eshima N Tokumaru O Hara S Bacal K Korematsu S Tabata M Sex- and age-related differences in morbidity rates of 2009 pandemic influenza A H1N1 virus of swine origin in Japan. PLoS One (2011) 6(4):e19409. 10.1371/journal.pone.0019409 21559366 PMC3084848

[32] Krementsov DN Case LK Dienz O Raza A Fang Q Ather JL Genetic variation in chromosome Y regulates susceptibility to influenza A virus infection. Proc Natl Acad Sci U S A (2017) 114:3491–6. 10.1073/pnas.1620889114 28242695 PMC5380050

[33] Straface E Gambardella L Brandani M Malorni W Sex differences at cellular level: “cells have a sex”. Handb Exp Pharmacol (2012) 214:49–65. 10.1007/978-3-642-30726-3_3 23027445

[34] Matarrese P Colasanti T Ascione B Margutti P Franconi F Alessandri C Gender disparity in susceptibility to oxidative stress and apoptosis induced by autoantibodies specific to RLIP76 in vascular cells. Antioxid Redox Signal (2011) 15:2825–36. 10.1089/ars.2011.3942 21671802

[35] Malorni W Straface E Matarrese P Ascione B Coinu R Canu S Redox state and gender differences in vascular smooth muscle cells. FEBS Lett (2008) 582:635–42. 10.1016/j.febslet.2008.01.034 18242172

[36] Straface E Vona R Campesi I Franconi F. Mitochondria can orchestrate sex differences in cell fate of vascular smooth muscle cells from rats. Biol Sex Differ (2015) 16(6):34. 10.1186/s13293-015-0051-9 26677409 PMC4681081

[37] Straface E Malorni W Pietraforte D Sex differences in redox biology: a mandatory new point of view approaching human inflammatory diseases. Antioxid Redox Signal (2017) 26:44–5. 10.1089/ars.2016.6931 27796122

[38] Watanabe H Numata K Ito T Takagi K Matsukawa A. Innate immune response in Th1- and Th2-dominant mouse strains. Shock (2004) 22:460–6. 10.1097/01.shk.0000142249.08135.e9 15489639

[39] Tate MD Brooks AG Reading PC. The role of neutrophils in the upper and lower respiratory tract during influenza virus infection of mice. Respir Res (2008) 9:57. 10.1186/1465-9921-9-57 18671884 PMC2526083

[40] Shirey KA Lai W Scott AJ Lipsky M Mistry P Pletneva LM The TLR4 antagonist Eritoran protects mice from lethal influenza infection. Nature (2013) 497:498–502. 10.1038/nature12118 23636320 PMC3725830

[41] Maxeiner JH Karwot R Hausding M Sauer KA Scholtes P Finotto S. A method to enable the investigation of murine bronchial immune cells, their cytokines and mediators. Nat Protoc (2007) 2:105–12. 10.1038/nprot.2007.8 17401344

[42] Conti G Magliani W Conti S Nencioni L Sgarbanti R Palamara AT Therapeutic activity of an anti-idiotypic antibody-derived killer peptide against influenza A virus experimental infection. Antimicrob Agents Chemother (2008) 52:4331–7. 10.1128/AAC.00506-08 18824612 PMC2592895

[43] Hu Q Zuo P Shao B Yang S Xu G Lan F Administration of nonviral gene vector encoding rat *β*-defensin-2 ameliorates chronic *Pseudomonas aeruginosa* lung infection in rats. J Gene Med (2010) 12:276–86. 10.1002/jgm.1435 20131335

[44] Lee-Lewis H Anderson DM. Absence of inflammationand pneumonia during infection with nonpigmented *Yersinia pestis* reveals a new role for the pgm locus in pathogenesis. Infect Immun (2010) 78:220–30. 10.1128/IAI.00559-09 19841077 PMC2798233

[45] Litzlbauer HD Neuhaeuser C Moell A Greschus S Breithecker A Franke FE Three-dimensional imaging and morphometry of alveolar tissue from microfocal x-ray-computed tomography. Am J Physiol Lung Cell Mol Physiol (2006) 291:L535–45. 10.1152/ajplung.00088.2005 16679382

[46] Carpino G Morini S Ginanni Corradini S Franchitto A Merli M Siciliano M Alpha-SMA expression in hepatic stellate cells and quantitative analysis of hepatic fibrosis in cirrhosis and in recurrent chronic hepatitis after liver transplantation. Dig Liver Dis (2005) 37:349–56. 10.1016/j.dld.2004.11.009 15843085

[47] Mouded M Egea EE Brown MJ Hanlon SM Houghton AM Tsai LW Epithelial cell apoptosis causes acute lung injury masquerading as emphysema. Am J Respir Cell Mol Biol (2009) 41:407–14. 10.1165/rcmb.2008-0137OC 19188661 PMC2746987

[48] Ellman GL Tissue sulfhydryl groups. Arch Biochem Biophys (1959) 82:70–7. 10.1016/0003-9861(59)90090-6 13650640

[49] Larcombe AN Foong RE Bozanich EM Berry LJ Garratt LW Gualano RC Sexual dimorphism in lung function responses to acute influenza A infection. Influenza Other Respir Viruses (2011) 5:334–42. 10.1111/j.1750-2659.2011.00236 21668688 PMC4942045

[50] Meister A Glutathione biosynthesis and its inhibition. Methods Enzymol (1995) 252:26–30. 10.1016/0076-6879(95)52005-8 7476360

[51] Klein SL. The effects of hormones on sex differences in infection: from genes to behavior. Neurosci Biobehav Rev (2000) 24:627–38. 10.1016/S0149-7634(00)00027-0 10940438

[52] Torcia MG Nencioni L Clemente AM Civitelli L Celestino I Limongi D Sex differences in the response to viral infections: TLR8 and TLR9 ligand stimulation induce higher IL10 production in males. PLoS One (2012) 7:e39853. 10.1371/journal.pone.0039853 22768144 PMC3387221

[53] Fukushi M Ito T Oka T Kitazawa T Miyoshi-Akiyama T Kirikae T Serial histopathological examination of the lungs of mice infected with influenza A virus PR8 strain. PLoS One (2011) 6:e21207. 10.1371/journal.pone.0021207 21701593 PMC3118813

[54] Kobasa D Jones SM Shinya K Kash JC Copps J Ebihara H Aberrant innate immune response in lethal infection of macaques with the 1918 influenza virus. Nature (2007) 445:319–23. 10.1038/nature05495 17230189

[55] Wareing MD Lyon AB Lu B Gerard C Sarawar SR. Chemokine expression during the development and resolution of a pulmonary leukocyte response to influenza A virus infection in mice. J Leukoc Biol (2004) 76:886–95. 10.1189/jlb.1203644 15240757

[56] Straub RH. The complex role of estrogens in inflammation. Endocr Rev (2007) 28:521–74. 10.1210/er.2007-0001 17640948

[57] Vom Steeg LG Vermillion MS Hall OJ Alam O McFarland R Chen H Age and testosterone mediate influenza pathogenesis in male mice. Am J Physiol Lung Cell Mol Physiol (2016) 311:L1234–44. 10.1152/ajplung.00352.2016 27815260 PMC5206399

[58] Du L Bayir H Lai Y Zhang X Kochanek PM Watkins SC Innate gender-based proclivity in response to cytotoxicity and programmed cell death pathway. J Biol Chem (2004) 279:38563–70. 10.1074/jbc.M405461200 15234982

[59] Fra A Yoboue ED Sitia R. Cysteines as redox molecular switches and targets of disease. Front Mol Neurosci (2017) 10:167. 10.3389/fnmol.2017.00167 28634440 PMC5459893

[60] Dickinson DA Forman HJ. Glutathione in defense and signaling: lessons from a small thiol. Ann N Y Acad Sci (2002) 973:488–504. 10.1111/j.1749-6632.2002.tb04690.x 12485918

[61] Fang YZ Yang S Wu G Free radicals, antioxidants, and nutrition. Nutrition (2002) 18(10):872–9. 10.1016/S0899-9007(02)00916-4 12361782

[62] Pajović SB Saicić ZS. Modulation of antioxidant enzyme activities by sexual steroid hormones. Physiol Res (2008) 57(6):801–11. 18052675 10.33549/physiolres.931377

[63] Massafra C Gioia D De Felice C Picciolini E De Leo V Bonifazi M Effects of estrogens and androgens on erythrocyte antioxidant superoxide dismutase, catalase and glutathione peroxidase activities during the menstrual cycle. J Endocrinol (2000) 167:447–52. 10.1677/joe.0.1670447 11115771

[64] Massafra C Gioia D De Felice C Muscettola M Longini M Buonocore G Gender-related differences in erythrocyte glutathione peroxidase activity in healthy subjects. Clin Endocrinol (Oxf) (2002) 57:663–7. 10.1046/j.1365-2265.2002.01657.x 12390342

[65] Urata Y Ihara Y Murata H Goto S Koji T Yodoi J 17Beta-estradiol protects against oxidative stress-induced cell death through the glutathione/glutaredoxin-dependent redox regulation of Akt in myocardiac H9c2 cells. J Biol Chem (2006) 281(19):13092–102. 10.1074/jbc.M601984200 16549430

[66] Chignalia AZ Oliveira MA Debbas V Dull RO Laurindo FR Touyz RM Testosterone induces leucocyte migration by NADPH oxidase-driven ROS- and COX2 dependent mechanisms. Clin Sci (2015) 129:39–48. 10.1042/CS20140548 25700020

[67] Reed DK Arany I. Sex hormones differentially modulate STAT3-dependent antioxidant responses during oxidative stress in renal proximal tubule cells. In Vivo (2014) 28:1097–100. 25398805

[68] Uchide N Toyoda H. Antioxidant therapy as a potential approach to severe influenza-associated complications. Molecules (2011) 16(3):2032–52. 10.3390/molecules16032032 21358592 PMC6259602

[69] Sgarbanti R Amatore D Celestino I Marcocci ME Fraternale A Ciriolo MR Intracellular redox state as target for anti-influenza therapy: are antioxidants always effective? Curr Top Med Chem (2014) 14(22):2529–41. 10.2174/1568026614666141203125211 25478883 PMC4435240

[70] Botta G Bizzarri BM Garozzo A Timpanaro R Bisignano B Amatore D Carbon nanotubes supported tyrosinase in the synthesis of lipophilic hydroxytyrosol and dihydrocaffeoyl catechols with antiviral activity against DNA and RNA viruses. Bioorg Med Chem (2015) 23(17):5345–51. 10.1016/j.bmc.2015.07.061 26260341 PMC7125559

[71] Aggarwal BB Deb L Prasad S. Curcumin differs from tetrahydrocurcumin for molecular targets, signaling pathways and cellular responses. Molecules (2014) 20(1):185–205. 10.3390/molecules20010185 25547723 PMC6272158

[72] Di Sotto A Checconi P Celestino I Locatelli M Carissimi S De Angelis M Antiviral and antioxidant activity of a hydroalcoholic extract from *Humulus lupulus* L. Oxidat Med Cell Long (2018). 10.1155/2018/5919237 PMC6081516 30140367

[73] Saladino R Neri V Checconi P Celestino I Nencioni L Palamara AT Synthesis of 2’-deoxy-1’-homo-N-nucleosides with anti-influenza activity by catalytic methyltrioxorhenium (MTO)/H2O2 oxyfunctionalization. Chemistry (2013) 19:2392–404. 10.1002/chem.201201285 23225323

[74] Bizzarri BM Botta L Capecchi E Celestino I Checconi P Palamara AT Regioselective IBX mediated synthesis of coumarin derivatives with antioxidant and anti-influenza activities. J Nat Prod (2017) 80(12):3247–54. 10.1021/acs.jnatprod.7b00665 29236486

[75] Bozzini T Botta G Delfino M Onofri S Saladino R Amatore D Tyrosinase and Layer-by-Layer supported tyrosinases in the synthesis of lipophilic catechols with antiinfluenza activity. Bioorg Med Chem (2013) 21(24):7699–708. 10.1016/j.bmc.2013.10.026 24216089

[76] Fioravanti R Celestino I Costi R Cuzzucoli Crucitti G Pescatori L Mattiello L Effects of polyphenol compounds on influenza A virus replication and definition of their mechanism of action. Bioorg Med Chem (2012) 20:5046–52. 10.1016/j.bmc.2012.05.062 22743086

[77] Yan Z Banerjee R. Redox remodeling as an immunoregulatory strategy. Biochemistry (2010) 49:1059–66. 10.1021/bi902022n 20070126 PMC2820725

[78] Dröge W Breitkreutz R Glutathione and immune function. Proc Nutr Soc (2000) 59:595–600. 10.1017/S0029665100000847 11115795

[79] Ghezzi P Role of glutathione in immunity and inflammation in the lung. Int J Gen Med (2011) 4:105–13. 10.2147/IJGM.S15618 21403800 PMC3048347

[80] Angelini G Gardella S Ardy M Ciriolo MR Filomeni G Di Trapani G Antigen-presenting dendritic cells provide the reducing extracellular microenvironment required for T lymphocyte activation. Proc Natl Acad Sci U S A (2002) 99(3):1491–6. 10.1073/pnas.022630299 11792859 PMC122218

[81] Mirkovic N Voehringer DW Story MD McConkey DJ McDonnell TJ Meyn RE. Resistance to radiation-induced apoptosis in Bcl-2-expressing cells is reversed by depleting cellular thiols. Oncogene (1997) 15:1461–70. 10.1038/sj.onc.1201310 9333022

[82] Voehringer DW McConkey DJ McDonnell TJ Brisbay S Meyn RE. Bcl-2 expression causes redistribution of glutathione to the nucleus. Proc Natl Acad Sci U S A (1998) 95:2956–60. 10.1073/pnas.95.6.2956 9501197 PMC19676

[83] Matarrese P Nencioni L Checconi P Ciarlo L Gambardella L Ascione B Pepstatin A alters host cell autophagic machinery and leads to a decrease in influenza A virus production. J Cell Physiol (2011) 226:3368–77. 10.1002/jcp.22696 21344392

[84] Lista P Straface E Brunelleschi S Franconi F Malorni W. On the role of autophagy in human diseases: a gender perspective. J Cell Mol Med (2011) 15:1443–57. 10.1111/j.1582-4934.2011.01293.x 21362130 PMC3823190

[85] Regitz-Zagrosek V Seeland U Sex and gender differences in clinical medicine. Handb Exp Pharmacol (2012) 214:3–22. 10.1007/978-3-642-30726-3_1 23027443

