# Supplementary figures and images for: Differential Redox State Contributes to Sex Disparities in the Response to Influenza Virus Infection in Male and Female Mice
Source: Front Immunol. 2018 Jul 30;9:1747. doi: 10.3389/fimmu.2018.01747 (PMC6077261; doi:10.3389/fimmu.2018.01747)

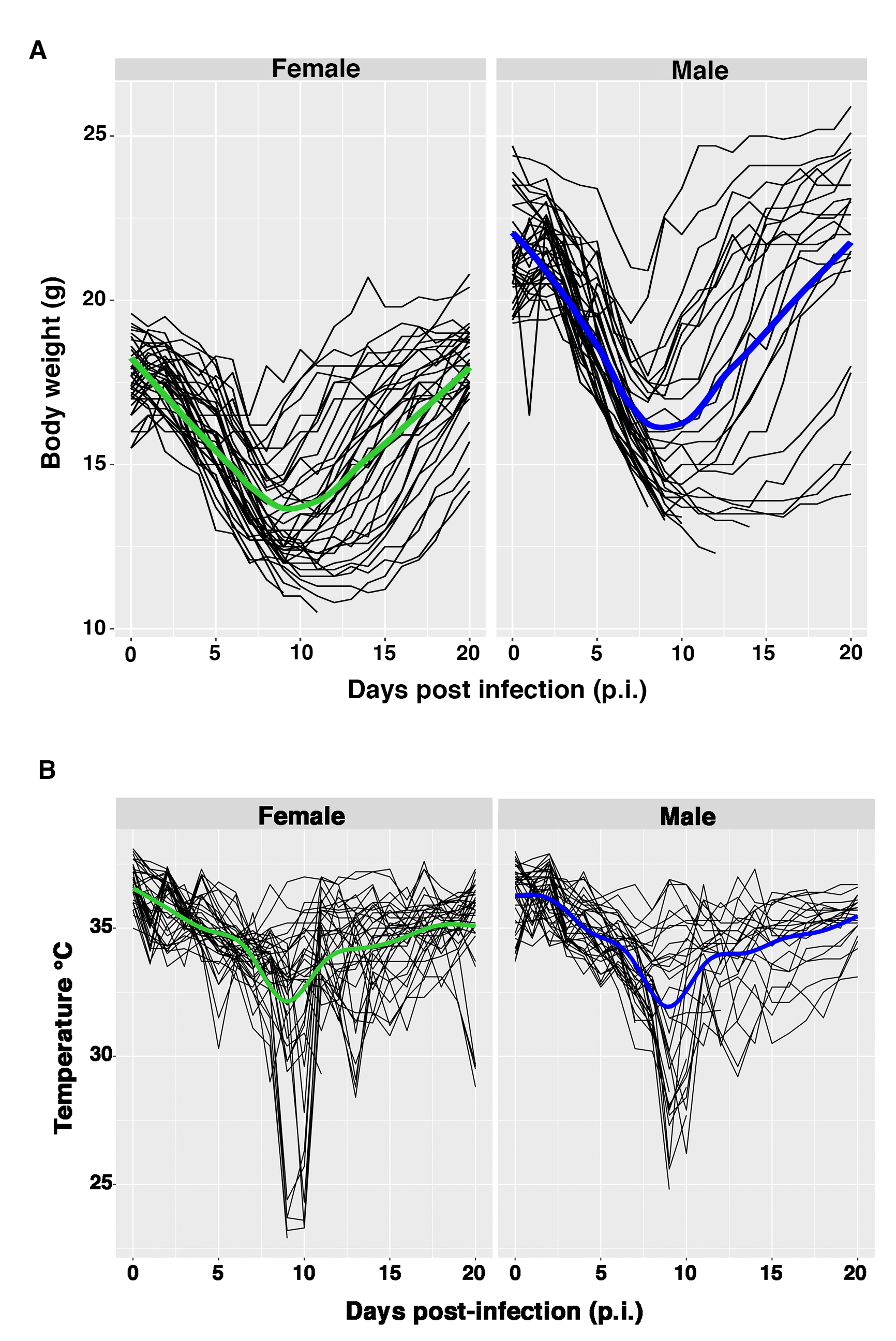

Supplement: Figure S1 — Body weight (bw) and temperature in female and male infected mice. (A) Spaghetti plot of the daily bw (expressed in grams); the bold lines represent the overall trend. (B) Spaghetti plot of the daily body temperature, the bold lines represent the overall trend. Results represent data pooled from 4 independent experiments, each performed with 10 males and 10 females (n = 40/sex). [file Image_1.tif]

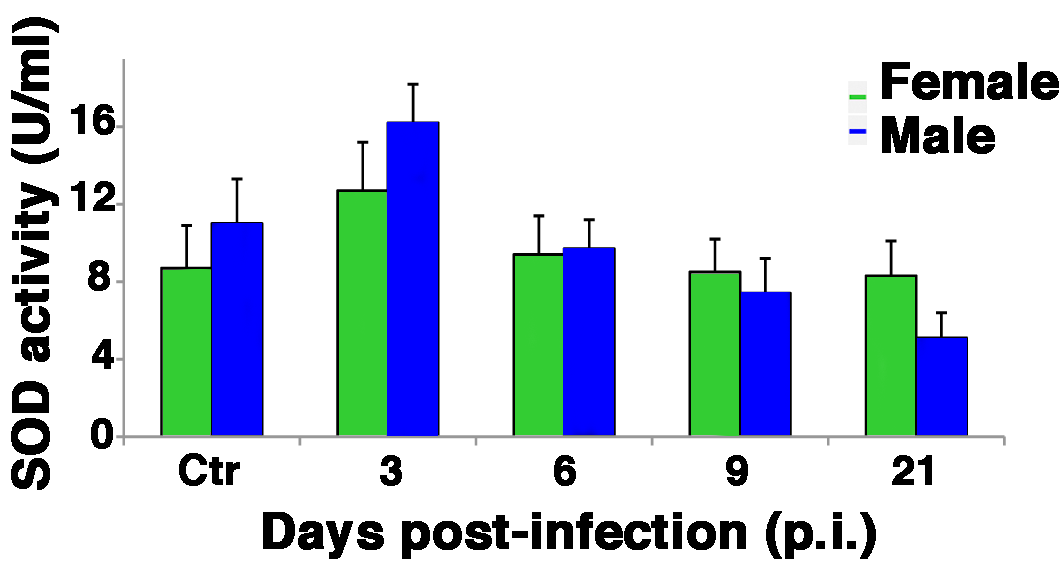

Supplement: Figure S2 — No differences in superoxide dismutase (SOD) activity were observed in infected female and male mice. Lung homogenates from Ctr and PR8-infected mice were assayed for SOD activity on p.i. days 3, 6, 9, and 21. Each value reported represents the mean ± SD of results from two separate experiments, each performed in duplicate (n = 4). [file Image_2.tiff]

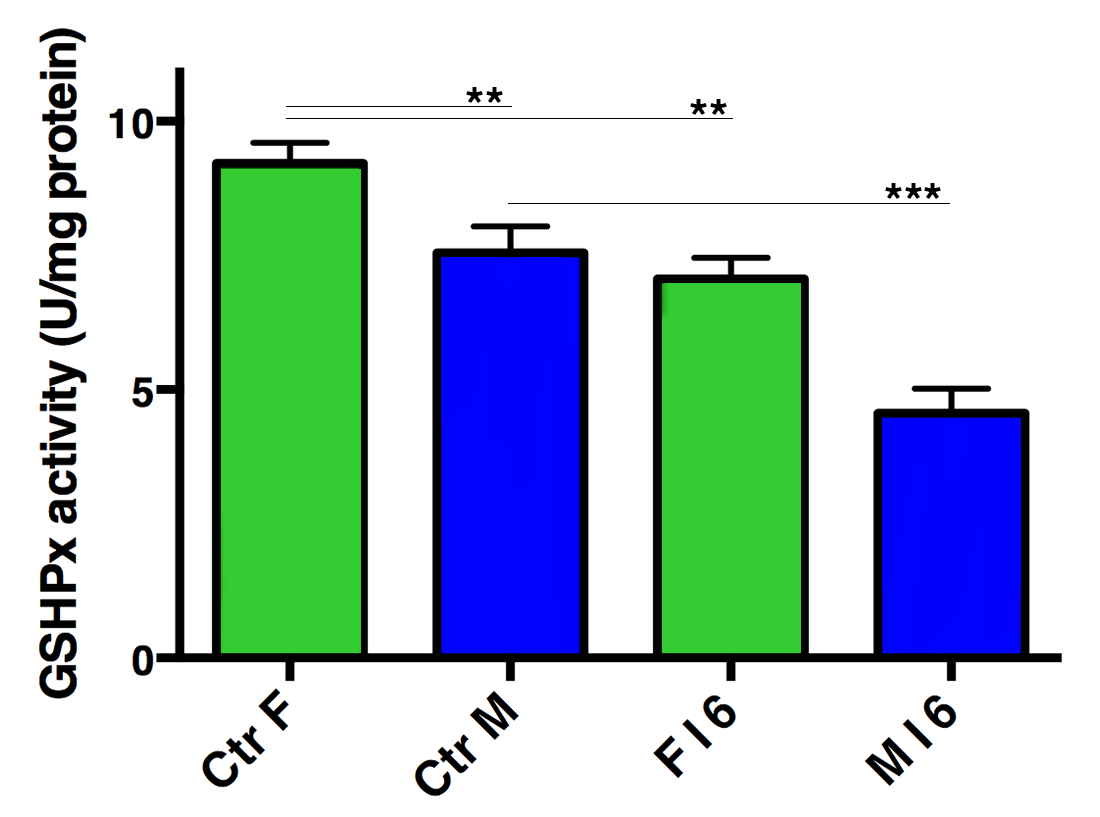

Supplement: Figure S3 — Glutathione peroxidase (GSHPx) activity is less reduced in infected female mice. Lung homogenates from Ctr and PR8-infected mice were assayed for GSHPx activity on p.i. day 6. Each value reported represents the mean ± SD of results from 3 mice/sex, each performed in triplicate (n = 9). One-way ANOVA test **p-value <0.01; ***p-value <0.001. [file Image_3.tif]
